# Counterbalancing O‐GlcNAcylation and STAT3 Phosphorylation in Ventral Tegmental Area Dopaminergic Neurons Mediates Behavioral Adaptations to Acute Restraint Stress

**DOI:** 10.1002/advs.202502701

**Published:** 2025-07-21

**Authors:** Mingshuo Shao, Yi Wu, Haiyang Wang, Chenchun Zhang, Ying Zhu, Yan Jiang, Changyou Jiang, Qiumin Le, Xing Liu, Lan Ma, Feifei Wang

**Affiliations:** ^1^ School of Basic Medical Sciences State Key Laboratory of Brain Function and Disorders MOE Frontiers Center for Brain Science, and Institutes of Brain Science Department of Neurosurgery Huashan Hospital Fudan University Shanghai 200032 China; ^2^ Research Unit of Addiction Memory Chinese Academy of Medical Sciences (2021RU009) Shanghai 200032 China

**Keywords:** dopaminergic neurons, emotion, post‐translational modifications, STAT3, stress

## Abstract

Acute stressors, significant risk factors for mood disorders, increase anxiety and reduce reward sensitivity. However, the underlying neuroadaptive mechanisms remain unclear. Here, it is shown that acute restraint stress upregulates Signal Transducer and Activator of Transcription 3 (STAT3) signaling and O‐GlcNAcylation in ventral tegmental area (VTA) dopaminergic (DAergic) neurons. Multi‐omics analyses in O‐linked N‐acetylglucosamine transferase (OGT) conditional knockout (cKO) mice reveal a counterbalancing mechanism between O‐GlcNAcylation and STAT3^Ser727^ phosphorylation. In vivo fiber‐optic recordings and behavioral experiments demonstrate that STAT3^Ser727^ phosphorylation mediates the suppression of DAergic neuronal activity and reward sensitivity, as well as the increased anxiety induced by acute restraint stress. Conversely, the upregulation of O‐GlcNAcylation in the VTA DAergic neurons prevents these effects. Furthermore, *Gabbr2* and *Gabrb3* expression is upregulated in the VTA of *Ogt* cKO mice, whereas the phosphorylation of STAT3^Ser727^ in the DAergic neurons is required for the upregulation of *Gabbr2* and *Gabrb3* induced by acute restraint stress. These findings highlight the dynamic and counterbalancing post‐translational modifications occurring in the VTA to maintain dopaminergic and emotional homeostasis, offering new insights into neuronal and behavioral responses to acute stress.

## Graphical abstract

Acute restraint stress enhances STAT3^Ser727^ phosphorylation in dopaminergic neurons in mice. This modification promotes the upregulation of GABBR2 and GABRB3, reduces neuronal activity, and contributes to anxiety‐like behavior and diminished reward‐seeking following stress exposure. Meanwhile, O‐GlcNAcylation is upregulated in the VTA, counteracting STAT3 phosphorylation and mitigating stress‐induced neuronal and behavioral changes to help maintain emotional homeostasis.

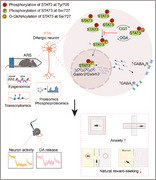

## 1 Introduction

Stress responses can be broadly categorized into acute and chronic forms, each inducing distinct patterns of neural adaptation.^[^ ^1^ ^]^ Acute stress triggers a rapid cascade of complex physiological and psychological responses and often results in heightened anxiety^[^ ^2^, ^3^ ^]^ and reduced reward sensitivity.^[^ ^4^, ^5^ ^]^ In contrast, chronic stress—characterized by prolonged or repeated exposure—leads to persistent neurobiological alterations that underlie emotional disturbances such as anxiety and depression.^[^ ^6^, ^7^ ^]^ The mesolimbic dopamine circuit originating in the ventral tegmental area (VTA) plays a crucial role in regulating dopamine (DA) release, thereby influencing emotional regulation and reward processing.^[^ ^8^, ^9^ ^]^ Acute stressors—including restraint,^[^ ^10^ ^]^ footshock,^[^ ^11^ ^]^ and pain^[^ ^12^ ^]^ —can significantly alter the activity patterns of the dopaminergic (DAergic) neurons,^[^ ^13^ ^]^ resulting in dysregulated DA release,^[^ ^14^ ^]^ which has been closely associated with the development of stress‐induced behavioral abnormalities.^[^ ^15^, ^16^ ^]^ Stress adaptation mechanisms are engaged to counteract the negative effects of stress and maintain emotional homeostasis.^[^ ^17^ ^]^ Neuronal signaling associated with these adaptations may underlie key biological processes contributing to resilience against stress‐related disorders. Understanding the activation of these intracellular signaling pathways in response to acute stress is essential for elucidating how stress reshapes the brain's reward system.

Numerous stress‐activated molecular pathways in the DAergic neurons facilitate recovery and adaptation to acute stress. Signal Transducer and Activator of Transcription 3 (STAT3), primarily activated by cytokines such as IL‐6 via the JAK/STAT pathway, serves as a critical mediator of neuronal adaptation to stress.^[^ ^18^ ^]^ It modulates transcriptional programs governing neuroprotection, synaptic plasticity, and inflammatory responses.^[^ ^19^, ^20^ ^]^ In DAergic neurons, STAT3 activity supports resilience by regulating oxidative stress and promoting anti‐apoptotic signaling.^[^ ^21^ ^]^ In addition to its canonical nuclear role, STAT3 also regulates mitochondrial function, linking extracellular stress to intracellular energy and redox balance.^[^ ^22^ ^]^ Its activity is tightly regulated through post‐translational modifications (PTMs), enabling the responses to acute oxidative stress.^[^ ^23^ ^]^ As a convergence point for multiple signaling pathways, including PI3K/AKT and MAPK/ERK, STAT3 plays a central role in neural homeostasis, with its dysregulation implicated in neuroinflammatory and neurodegenerative disorders.^[^ ^24^ ^]^

O‐linked β‐N‐acetylglucosamine (O‐GlcNAc) glycosylation is a dynamic PTM regulated by O‐GlcNAc transferase (OGT) and O‐GlcNAcase (OGA).^[^ ^25^ ^]^ This modification antagonizes phosphorylation at adjacent serine/threonine residues,^[^ ^26^ ^]^ and plays a pivotal role in modulating cellular stress responses.^[^ ^27^ ^]^ O‐GlcNAcylation contributes to stress adaptation by regulating processes at both transcriptional and post‐translational levels,^[^ ^28^ ^]^ thereby fine‐tuning cellular mechanisms essential for survival and resilience. O‐GlcNAcylation has emerged as a key modulator of STAT3 post‐translational regulation, particularly through its interaction with phosphorylation at specific residues. Studies have shown that increased O‐GlcNAcylation correlates with elevated phosphorylation at STAT3^Tyr705^ and reduced phosphorylation at STAT3^Ser727^, suggesting a site‐specific antagonism under high‐glucose conditions.^[^ ^29^ ^]^ Additionally, loss of O‐GlcNAc modification at STAT3^Thr717^ has been linked to altered STAT3 transcriptional activity in neural stem cells.^[^ ^30^ ^]^ These findings highlight a dynamic crosstalk between O‐GlcNAcylation and phosphorylation in fine‐tuning STAT3 function.

In this study, we investigated the functional interaction between O‐GlcNAcylation and STAT3 phosphorylation in the VTA in response to acute restraint stress. The phosphorylation of STAT3 regulates its transcriptional activity in response to various stressors. However, how this modification contributes to neuronal and behavioral adaptations during acute restraint stress, and whether O‐GlcNAcylation functions as a stress‐buffering mechanism by antagonizing STAT3 phosphorylation to prevent neuronal and behavioral disturbances, remains to be investigated. These observations may help uncover a previously unrecognized regulatory axis integrating PTMs, transcription, and neuronal signaling in the mesolimbic dopamine system to maintain emotional homeostasis.

## 2 Results

### 2.1 Acute Restraint Stress Upregulates STAT3 Signaling and O‐GlcNAcylation in the VTA DAergic Neurons

**Figure 1:**
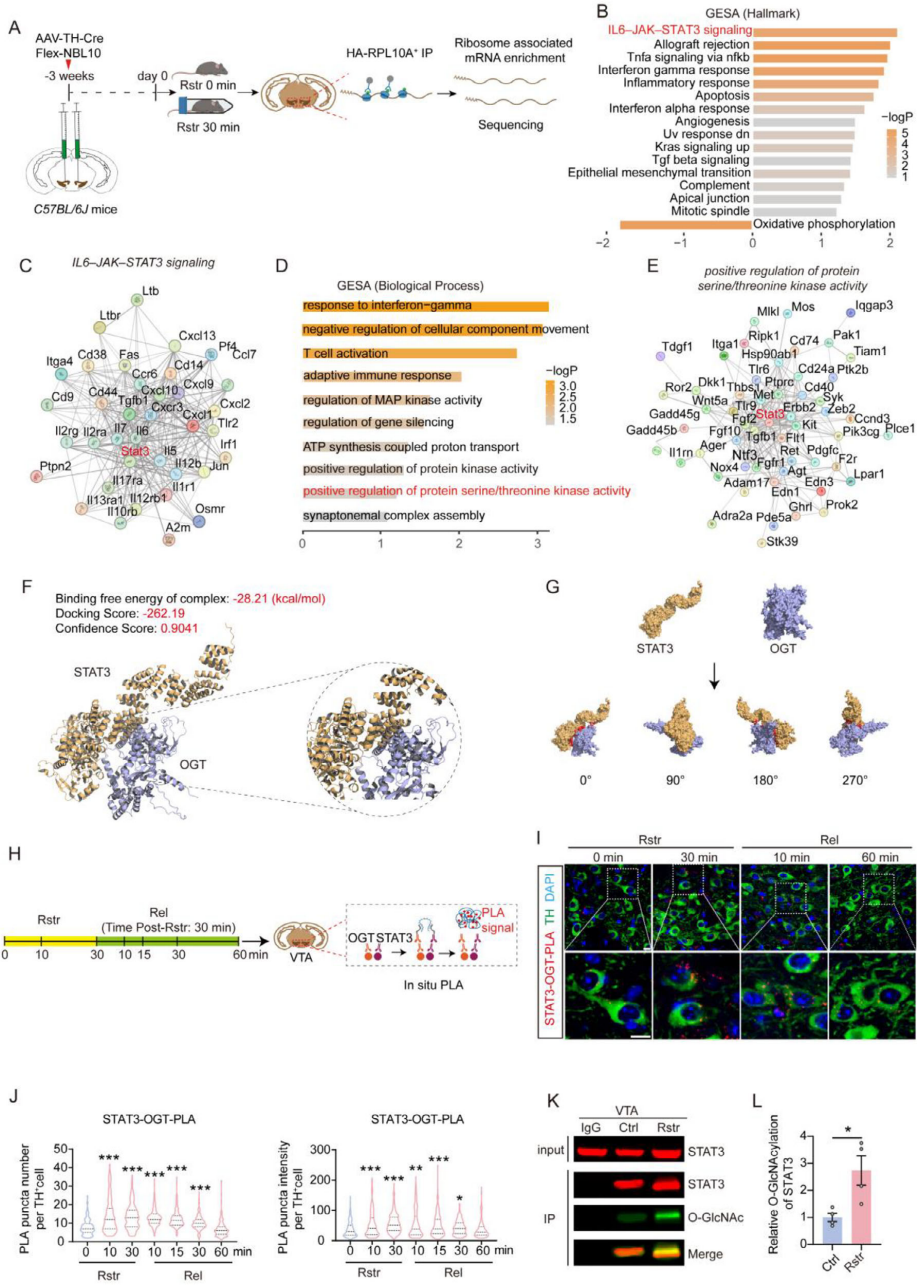
Acute restraint stress enhances IL6–JAK–STAT3 signaling and promotes STAT3 O‐GlcNAcylation in the VTA DAergic neurons. A) Schematic of the experimental procedure for isolating ribosome‐associated mRNA from VTA DAergic neurons and performing RNA‐Seq in male C57BL/6J mice subjected to 30 min of restraint or homecage control. *n* = 5 samples from 10 mice per group. B) GSEA of gene transcripts altered by 30 min of restraint in DAergic neurons. C) Protein interaction network between genes significantly altered in the IL6–JAK–STAT3 signaling pathway. Nodes represent proteins, and edges depict interactions between them. D) GO biological process enrichment analysis of differentially expressed transcripts in DAergic neurons. GO terms are ranked by enrichment score. E) Protein interaction network of genes significantly involved in the positive regulation of protein serine/threonine kinase activity signaling pathway. F) Molecular docking of OGT and STAT3 performed using HDOCK. STAT3 is shown in yellow and OGT in purple. G) Surface representation of the OGT–STAT3 docking structure. STAT3 is shown in yellow, OGT in purple, and the protein‐protein interaction interface in red. H) Schematic illustration of in situ PLA detection of OGT–STAT3 interactions in VTA DAergic neurons.VTA tissues were collected from mice restrained for 0, 10, and 30 min, and from mice released for 10, 15, 30, and 60 min after 30 min of restraint. I,J) Representative confocal images (I) and quantitative analysis of the mean number and mean intensity of PLA puncta per TH^+^ cell in the VTA (J). *n* = 240 cells from 6 mice per group. Green: TH; Red: PLA puncta; Blue: DAPI. Scale bar: 20 µm. K,L) STAT3 in the VTA was immunoprecipitated (K), and analyzed using an anti‐O‐GlcNAcylation antibody (L). *n* = 4 samples from 8 mice per group. Data are presented as mean ± SEM. ^*^ *p* < 0.05, ^**^ *p* < 0.01, ^***^ *p* < 0.001; *p*‐values are calculated using Kruskal–Wallis H test with *Bonferroni post hoc* test (J), or Welch's t‐test (L).

To investigate the acute stress‐induced transcriptional changes in VTA DAergic neurons, ribosome‐associated transcripts were isolated from VTA DAergic neurons in mice that had been subjected to either 30 min of acute restraint stress or a control homecage condition (**Figures** **1**A, and S1A, Supporting Information). Differentially expressed genes (DEGs) were identified after RNA sequencing, including 144 upregulated and 201 downregulated genes (Figure S1B, Supporting Information). Gene Ontology (GO) and Kyoto Encyclopedia of Genes and Genomes (KEGG) enrichment analyses indicated significant enrichment in PTM‐related pathways, such as histone acetylation and oxidative phosphorylation (Figure S1C–E, Supporting Information). Gene set enrichment analysis (GSEA) showed that the IL6‐JAK‐STAT3 signaling pathway was the most significantly upregulated in response to acute restraint stress (Figure 1B,C). Biological process enrichment and protein interaction analysis of DEGs involved in the positive regulation of protein serine/threonine kinase activity signaling pathways revealed that STAT3 was positioned centrally within the network (Figure 1D,E). Consistently, the expression of STAT3 downstream target genes—such as *Cxcl10, Osmr, Il6, Ccl7, Il1r1, and Ptpn2* ^[^ ^31^, ^32^, ^33^ ^]^ —was significantly elevated in response to acute restraint stress (Figure S1F, Supporting Information). These findings suggest the potential involvement of STAT3 phosphorylation regulation in DAergic neurons in response to acute restraint stress.

O‐GlcNAcylation, a stress‐responsive PTM regulated by OGT and OGA, antagonizes protein serine/threonine phosphorylation at adjacent sites.^[^ ^25^ ^]^ Protein–protein docking between OGT and STAT3 was performed using the HDOCK method, and the interaction was visualized using a surface representation. The docking and confidence scores indicated a high likelihood of binding between the two proteins (Figure 1F,G). The interaction between OGT and STAT3 in VTA DAergic (TH⁺) neurons—identified by the dopamine‐synthesizing enzyme tyrosine hydroxylase (TH)—was further assessed using proximity ligation assay (PLA) (Figure 1H). The number and intensity of PLA puncta signals in TH^+^ cells were significantly increased after 10 and 30 min of restraint stress and remained detectable for up to 30 min after release, indicating OGT‐STAT3 interactions in VTA DAergic neurons (Figure 1I,J). Furthermore, knockdown of *Ogt* in the VTA DAergic neurons markedly reduced PLA signals in TH⁺ cells to nearly undetectable levels (Figure S2, Supporting Information). To assess changes in STAT3 O‐GlcNAcylation induced by acute restraint stress, VTA tissues were collected from mice and subjected to STAT3 immunoprecipitation. Subsequent immunoblotting showed a significant increase in STAT3 O‐GlcNAcylation in the restrained group compared to controls (Figure 1K,L).

These results suggest that the increased interaction of OGT with STAT3 may play a role in the response of VTA DAergic neurons to acute restraint stress.

### 2.2 Knockdown of *Ogt* in VTA DAergic Neurons Activates STAT3 Signaling in Response to External Stimuli and Promotes STAT^Ser727^ Phosphorylation

**Figure 2:**
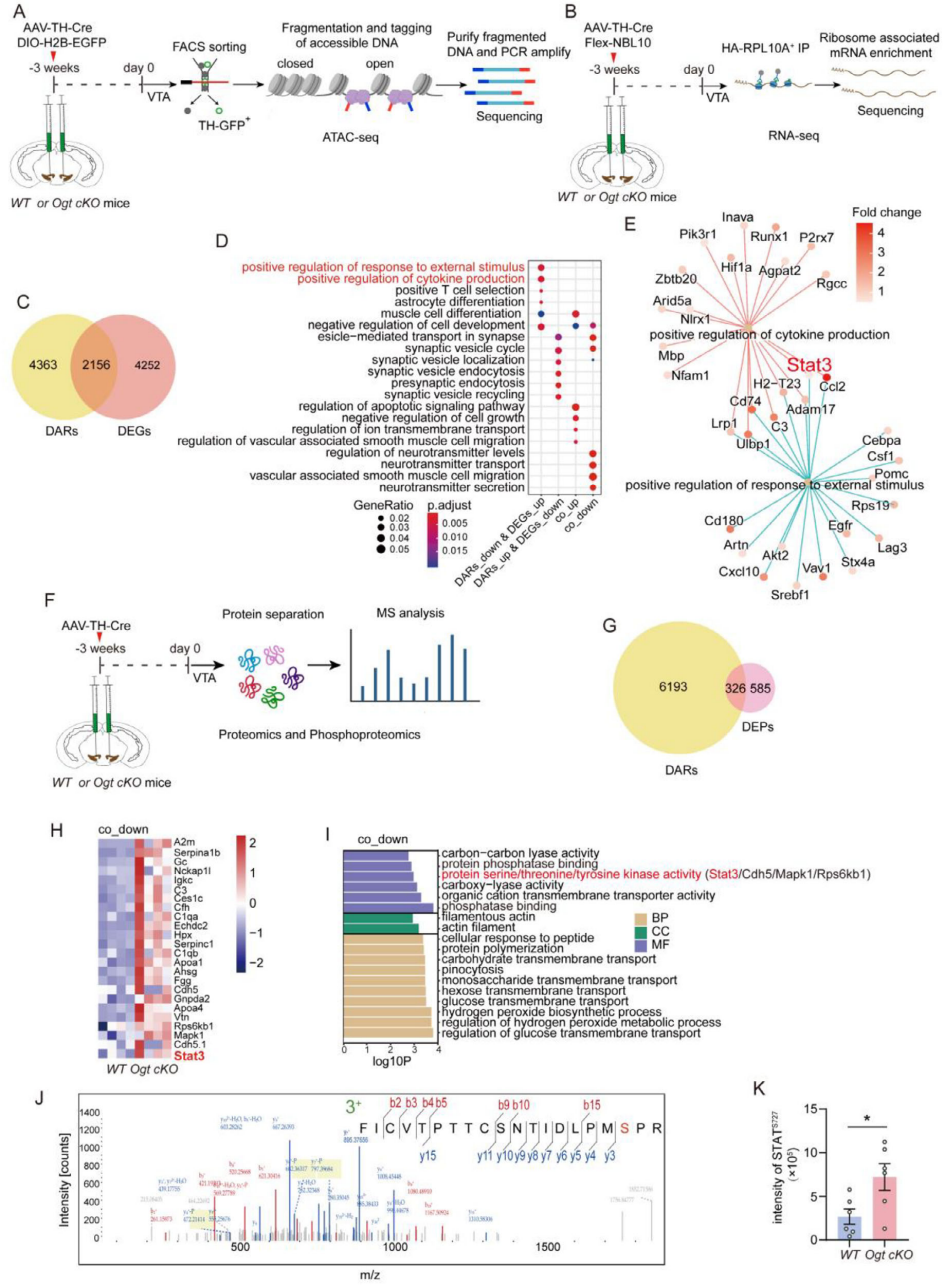
Knockdown of *Ogt* in VTA DAergic neurons activates upregulated signaling pathways in response to external stimuli and promotes STAT^Ser727^ phosphorylation. A,B) Schematic diagrams illustrating the experimental workflows for ATAC‐seq (A) and RNA‐seq (B) in the VTA DAergic neurons. For ATAC‐seq: *n* = 3 samples from 9 mice per group (*Ogt ^flox+/Y^* and *Ogt ^flox‐/Y^*). For RNA‐seq: *n* = 4 samples from 8 mice per group (*Ogt ^flox+/Y^* and *Ogt ^flox‐/Y^*). C) Venn diagram showing the overlap between DARs and DEGs. D) GO biological process enrichment analysis of genes overlapping with DARs and DEGs. Circle size represents the gene ratio, and color indicates the adjusted *p‐*value reflecting enrichment significance. E) Gene co‐expression network analysis of two biological processes: positive regulation of cytokine production and response to extracellular stimuli. Circle color represents gene expression fold change. F) Schematic diagram illustrating proteomic and phosphoproteomic analyses of VTA tissue from mice injected with *AAV‐TH‐Cre* into the VTA. *n* = 4 samples from 12 mice per group (*Ogt ^flox+/Y^* and *Ogt ^flox‐/Y^*). G) Venn diagram showing the overlap between DARs and DEPs. H) Heatmap illustrating the abundance patterns of DEPs in the co_down group. Each row represents a protein, with color intensity indicating fold change. I) GO enrichment analysis of molecular function (MF), cellular component (CC), and biological process (BP) terms for co_down genes overlapping with DARs and DEPs. J,K) Representative mass spectrometry spectra showing STAT phosphorylation sites (J), and quantification of STAT^Ser727^ phosphorylation intensity (K). *n* = 4 samples from 12 mice per group (*Ogt ^flox+/Y^* and *Ogt ^flox‐/Y^*). Data are presented as mean ± SEM. ^*^ *p* < 0.05; *p*‐values are calculated using Two‐tailed unpaired Student's t‐test (K).

To investigate the signaling pathways regulated by O‐GlcNAcylation, ATAC‐seq and RNA‐seq were performed on VTA DAergic neurons. *AAV‐TH‐Cre* and *AAV‐DIO‐H2B‐EGFP* were injected into the VTA of *Ogt cKO* (*Ogt ^flox+/Y^*) mice and their wild‐type (WT) littermates (*Ogt ^flox‐/Y^*) (**Figure** **2**A). EGFP^+^ nuclear fractions from the VTA of *Ogt cKO* and WT mice were subjected to ATAC‐seq analysis. A significant reduction in the number of EGFP⁺ nuclei was observed in *Ogt cKO* mice compared to their WT littermates, indicating that *Ogt* is essential for the survival of VTA DAergic neurons (Figure S3A,B, Supporting Information). For RNA‐seq analysis, *AAV‐TH‐Cre* and *AAV‐DIO‐NBL10‐HA* were delivered into the VTA of *Ogt cKO* and WT mice. Ribosome‐associated mRNA was isolated from VTA DAergic neurons and subjected to RNA sequencing (Figure 2B).

An overlap analysis of differentially accessible regions (DARs) and DEGs (Figure 2C; Figure S3C,D, Supporting Information) revealed significant enrichment in biological processes related to the positive regulation of responses to external stimuli and cytokine production in VTA DAergic neurons following *Ogt* knockdown (Figure 2D). Consistently, gene co‐expression network analysis identified STAT3 as a central transcription factor regulating these pathways (Figure 2E).

Proteomic analysis was performed on VTA tissues from *Ogt* cKO mice and their WT littermates (Figure 2F). Overlap analysis of DARs and differentially expressed proteins (DEPs) (Figure 2G, Figure S3E,F, Supporting Information) revealed a downregulation of STAT3 (Figure 2H). Furthermore, molecular function enrichment analysis indicated a reduction in protein serine/threonine kinase activity in *Ogt cKO* VTA tissues (Figure 2I). Phosphoproteomic analysis revealed an upregulation of STAT phosphorylation at the Ser727 site in *Ogt cKO* mice (Figure 2J,K).

*Ogt cKO* mice and their WT littermates were injected with *AAV‐TH‐Cre* and AAV vector encoding Cre‐dependent HA‐tagged STAT3 into the VTA (Figure S3G, Supporting Information). VTA tissues were isolated and subjected to STAT3‐specific chromatin immunoprecipitation (ChIP) using an anti‐HA antibody. The results showed that *Ogt* knockdown enhanced STAT3 enrichment at the upstream regions of transcription start sites (TSS) of several target genes, including *Cxcl10, Il6, Myc, Hif1a, and Bcl‐2* ^[^ ^34^, ^35^ ^]^ (Figure S3H, Supporting Information).

These results suggest that O‐GlcNAcylation modulates STAT3 phosphorylation and regulates its promoter binding activity in VTA DAergic neurons.

### 2.3 O‐GlcNAcylation Inhibits the Increased STAT3^Ser727^ Phosphorylation in VTA DAergic Neurons Induced by Acute Restraint Stress

**Figure 3:**
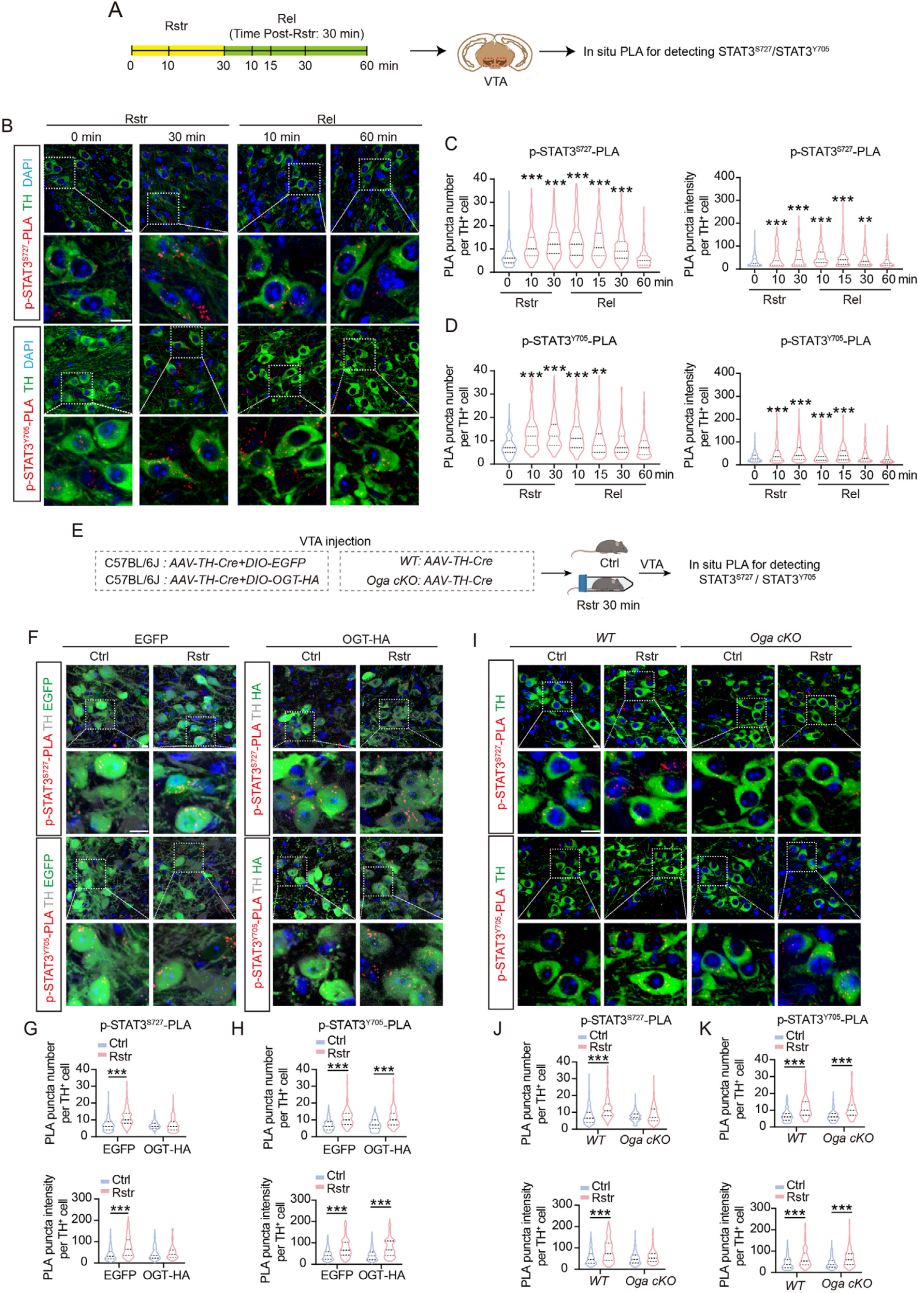
The increase in STAT3^Ser727^ phosphorylation in VTA DAergic neurons induced by acute restraint stress is suppressed by O‐GlcNAcylation. A) Schematic diagram illustrating the detection of STAT3 phosphorylation at Tyr705 and Ser727 in VTA DAergic neurons by in situ PLA. Brain tissues were collected from mice restrained for 0, 10, and 30 min, as well as from mice released for 10, 15, 30, and 60 min after 30 min of restraint. B–D) Representative confocal images (B) and quantitative analysis of the mean number and fluorescence intensity (C,D) of PLA puncta per TH^+^ cell. Green: TH; Red: PLA puncta of p‐STAT3^Ser727^ and p‐STAT3^Tyr705^; Blue: DAPI. Scale bar: 20 µm. *n* = 240 cells from 6 mice per group. E–K) Schematic diagram illustrating in situ PLA detection of STAT3 phosphorylation at Tyr705 or Ser727 in VTA DAergic neurons from mice with OGT overexpression (F–H) or OGA knockdown (I,K). Representative confocal images (F, I). F: Gray: TH; Green: EGFP/HA; Red: PLA puncta. I: Green: TH; Red: PLA puncta. Scale bar: 20 µm. Quantification of the mean number and fluorescence intensity of PLA puncta showing p‐STAT3^Ser727^ (G, J) and p‐STAT3^Tyr705^ (H, K) per TH^+^ cell. *n* = 240 cells from 6 mice per group. Data are presented as mean ± SEM. ^**^ *p* < 0.01, ^***^ *p* < 0.001; *p*‐values are calculated using Kruskal–Wallis H test with *Bonferroni post hoc* test (C‐D), or Scheirer–Ray–Hare test with *Bonferroni post hoc* test (G, H, J, K).

The phosphorylation levels of STAT3 at Ser727 (p‐STAT3^S727^) and Tyr705 (p‐STAT3^Y705^) in the VTA were evaluated. Immunoblotting analysis revealed that both p‐STAT3^S727^ and p‐STAT3^Y705^ were significantly upregulated after acute restraint stress, and returned to basal levels after release, while total STAT3 expression remained unchanged (Figure S4A–F, Supporting Information). Similarly, PLA puncta signals for p‐STAT3^S727^ and p‐STAT3^Y705^ in VTA TH^+^ cells were significantly increased 10 min after acute restraint stress and returned to baseline levels within 60 min after release (**Figure** **3**A–D). In contrast, total STAT3 PLA signals remained unchanged throughout these stages (Figure S4G,H, Supporting Information).

The hypothalamic–pituitary–adrenal (HPA) axis modulates VTA function via glucocorticoid signaling.^[^ ^36^ ^]^ Stress‐induced glucocorticoids act on VTA DAergic neurons to regulate their excitability and DA release.^[^ ^37^, ^38^ ^]^ Corticosterone levels in the serum increased significantly 10 min after restraint and returned to baseline 30 min after release, indicating a robust and dynamic activation of the HPA axis by acute restraint stress (Figure S4I,J, Supporting Information), in parallel with changes in the dynamics of stress‐induced STAT3 phosphorylation.

The effects of O‐GlcNAcylation upregulation on STAT3^S727^ and STAT3^Y705^ phosphorylation in VTA DAergic neurons induced by acute restraint stress were further investigated (Figure 3E). *AAV‐TH‐Cre* combined with a Cre‐dependent AAV encoding either HA‐tagged OGT (OGT group) or EGFP control (EGFP group) (Figure S5A–C, Supporting Information) was injected into the VTA of mice. In the EGFP group, acute restraint stress significantly increased p‐STAT3^S727^ fluorescence intensity and PLA puncta in VTA TH^+^ cells. In contrast, no significant changes in p‐STAT3^S727^ levels were observed in the OGT group under the same conditions, indicating that O‐GlcNAcylation upregulation inhibits STAT3^S727^ phosphorylation (Figure 3F,G). P‐STAT3^Y705^, which is not regulated by O‐GlcNAcylation, was significantly increased in both the OGT and EGFP groups after acute restraint stress (Figure 3F,H).

To further validate these findings, we generated *Oga* conditional knockout (*cKO*) (*Oga^flox+/+^*) mice (Figure S5D, Supporting Information). qRT‐PCR analysis confirmed the knockdown of *Oga* in ribosome‐associated transcripts isolated from VTA DAergic neurons (Figure S5E,F, Supporting Information). Immunostaining showed increased O‐GlcNAcylation levels in TH^+^ neurons of *Oga cKO* mice (Figure S5G–I, Supporting Information). In WT littermates (*Oga^flox‐/‐^*), acute restraint stress significantly increased the PLA puncta signal for p‐STAT3^S727^ levels in TH^+^ cells. In contrast, no significant changes in p‐STAT3^S727^ levels were observed in *Oga* cKO mice under the same conditions (Figure 3I,J). For p‐STAT3^Y705^, acute restraint stress induced a significant increase in PLA signals in both *Oga cKO* mice and WT littermates (Figure 3I,K).

These results suggest that acute restraint stress upregulates phosphorylation of STAT3^Ser727^ in VTA DAergic neurons, whereas protein O‐GlcNAcylation inhibits this phosphorylation.

### 2.4 STAT3^Ser727^ Phosphorylation Deficiency in VTA DAergic Neurons Impairs Acute Restraint Stress‐Induced Suppression of Neuronal Activity and DA Release

*Stat3* conditional knockout (*cKO*) mice (*Stat3^flox+/+^*) were generated to investigate the role of STAT3 in VTA DAergic neuron activity and DA release (Figure S6A, Supporting Information). Immunoblotting confirmed a reduction in STAT3 protein levels in VTA tissue (Figure S6B–D, Supporting Information). Additionally, qRT‐PCR analysis of ribosome‐associated transcripts validated the effective knockdown of *Stat3* in VTA DAergic neurons (Figure S6E,F, Supporting Information).

To monitor calcium dynamics in VTA DAergic neurons*, AAV‐TH‐Cre* and *AAV‐Flex‐jGCaMP7b* were injected into the VTA of *Stat3 cKO* and their WT littermates (*Stat3^flox‐/‐^*) (Figure S6G, Supporting Information). In WT mice, calcium signaling in VTA DAergic neurons decreased during acute restraint stress and remained low after release. In contrast, *Stat3 cKO* mice showed no significant decrease in calcium dynamics in response to acute restraint stress (Figure S6H–J, Supporting Information). Calcium dynamics were also examined during natural reward‐related behaviors, including sucrose licking (Figure S7A–C, Supporting Information) and social sniffing (Figure S7F–H, Supporting Information). No significant differences in calcium dynamics were observed between *Stat3 cKO* and WT mice during either behavior.

To assess DA release*, AAV‐TH‐Cre* was injected into the VTA of *Stat3 cKO* and their WT littermates, and fluorescence dynamics of the dopamine sensor DA4.4 were monitored in the nucleus accumbens (NAc) (Figure S6K, Supporting Information). In WT mice, DA4.4 signals in the NAc decreased significantly during restraint and remained low after release. In contrast, *Stat3 cKO* mice showed no such changes in DA4.4 signals during or after restraint (Figure S6L,N, Supporting Information). However, *Stat3* knockdown did not affect VTA DAergic neuronal activity or DA release during sucrose licking (Figure S7D,E, Supporting Information) or social sniffing (Figure S7I,J, Supporting Information). Additionally, in vivo PLA analysis revealed no change in STAT3^Ser727^ phosphorylation in VTA DAergic neurons following the consumption of freely available sugar pellets (Figure S7K,L, Supporting Information).

These results suggest that STAT3 in the VTA may selectively regulate DAergic neuron activity and DA release in response to acute restraint stress, while having minimal influence on their activity during natural reward‐related behaviors.

**Figure 4:**
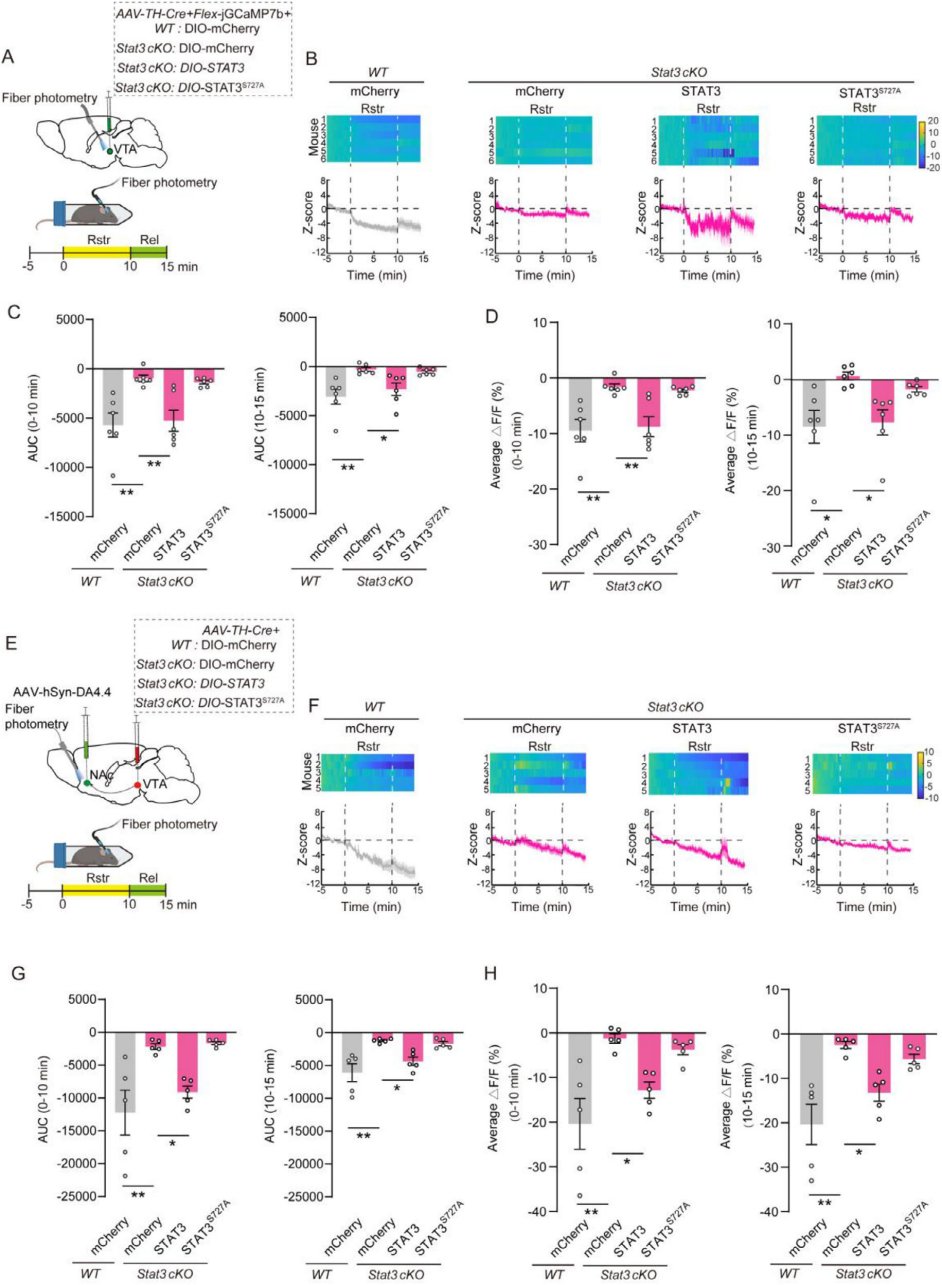
Deficiency of STAT3^Ser727^ phosphorylation in the VTA DAergic neurons abolishes the suppression of neuronal activity and DA release induced by acute restraint stress. A) Schematic diagram illustrating viral injection and fiber photometry recordings of calcium signals in VTA DAergic neurons before, during, and after restraint stress in *Stat3^flox+/+^* mice and their WT littermates. B) Heatmap (top) and Z‐score normalized average calcium signal responses (bottom) recording in the VTA DAergic neurons during acute restraint and release. Shaded areas represent SEM. *n* = 6 mice per group. C) Area under the curve (AUC) of calcium signal responses aligned to the onset of restraint (records from the 5 min before restraint were used as a baseline). D) Average peak calcium signal responses aligned to the onset of restraint and release. E) Schematic diagram illustrating viral injection and fiber photometry recordings of the DA4.4 sensor in the NAc before, during, and after restraint stress in *Stat3^flox+/+^* mice and their WT littermates. F) Heatmap (top) and Z‐score normalized average DA4.4 sensor responses (bottom) in the NAc during restraint and release. *n* = 5 mice per group. Shaded areas represent SEM. G) AUC of DA4.4 sensor responses aligned to the onset of restraint. H) Average peak of DA4.4 responses aligned to the onset of restraint and release. Data are presented as mean ± SEM. ^*^ *p* < 0.05, ^**^ *p* < 0.01; *p*‐values are calculated using One‐way ANOVA with *Bonferroni post hoc* test (C, G, H), Kruskal–Wallis H test with *Bonferroni post hoc* test (D) or Welch's ANOVA with *Bonferroni post hoc* test (G).

Cre‐dependent AAVs encoding HA‐tagged STAT3, the phosphorylation‐deficient mutant STAT3^S727A^, or the phosphomimetic mutant STAT3^S727D^ were generated and co‐delivered into the VTA with *AAV‐TH‐Cre* (Figure S8A,B, Supporting Information). The effects of STAT3^S727A^ and STAT3^S727D^ on STAT3^Ser727^ phosphorylation were assessed in VTA tissue. In situ PLA analysis revealed a significant decrease in p‐STAT3^Ser727^ in TH^+^ cells expressing STAT3^S727A^ and a marked increase in those expressing STAT3^S727D^, compared to the STAT3‐ expressing group (Figure S8C,D, Supporting Information). To investigate the role of STAT3^Ser727^ phosphorylation in DAergic neuron activity during acute restraint stress, *AAV‐TH‐Cre*, *AAV‐Flex‐jGCaMP7b*, and Cre‐dependent AAV encoding STAT3^S727A^ were co‐injected into the VTA of *Stat3 cKO* and WT mice (**Figure** **4**A). In WT littermates expressing mCherry, VTA DAergic neuron activity significantly decreased during restraint, whereas no such changes were observed in *Stat3 cKO* mice expressing mCherry (Figure 4B–D). Overexpression of STAT3 in *Stat3 cKO* mice restored the restraint‐induced inhibition of neuronal activity. However, this effect was not observed in mice overexpressing the STAT3^S727A^ mutant (Figure 4B–D).

The effect of STAT3^Ser727^ phosphorylation on DA release was assessed using DA4.4 dopamine sensors (Figure 4E). In WT mice expressing mCherry, DA release in the NAc significantly decreased during restraint and remained suppressed after release. In contrast, no inhibition of DA4.4 signals was observed in *Stat3 cKO* mice expressing mCherry (Figure 4F–H). Overexpression of STAT3 in VTA DAergic neurons restored the restraint‐induced suppression of DA release in *Stat3 cKO* mice. However, this effect was not observed in mice overexpressing the STAT3^S727A^ mutant (Figure 4F–H).

These results suggest that STAT3^Ser727^ phosphorylation in VTA DAergic neurons is required for the inhibition of neuronal activity and DA release in the NAc induced by acute restraint stress.

### 2.5 STAT3^Ser727^ Phosphorylation Deficiency in VTA DAergic Neurons Reverses Anxiety and Reward‐Seeking Suppression Induced by Acute Restraint Stress

Previous results showed that both the activity of DAergic neurons in the VTA and DA release in the NAc remained suppressed after release from restraint. To investigate the behavioral relevance of this effect and the role of STAT3, we conducted behavioral analyses after stress exposure. *AAV‐TH‐Cre* was injected into the VTA of *Stat3 cKO* mice and their WT littermates. Behavioral experiments were conducted on mice subjected to either 30 min of restraint or no restraint (Figure S9A, Supporting Information). In WT controls, acute restraint significantly reduced the time spent in the central area during the open field test (OFT) (Figure S9B–D, Supporting Information) and decreased both time in and entries into the open arms of the elevated plus maze (EPM) (Figure S9E–G, Supporting Information), indicating increased anxiety levels. In contrast, *Stat3 cKO* mice did not exhibit elevated anxiety after acute restraint stress.

In the social interaction test, WT littermates spent more time exploring the novel mouse than the empty cage, but this preference was abolished by acute restraint stress—a phenomenon not observed in *Stat3 cKO* mice (Figure S9H–J, Supporting Information). In the sucrose conditioned place preference (CPP) test, mice were subjected to restraint before each sucrose conditioning session (Figure S9K, Supporting Information). During the test session, WT mice showed significantly lower CPP scores compared to non‐restrained controls, indicating reduced sucrose preference after acute restraint stress. In contrast, social interaction and sucrose preference behaviors in *Stat3 cKO* mice remained unaffected by acute restraint stress (Figure S9L,M, Supporting Information).

**Figure 5:**
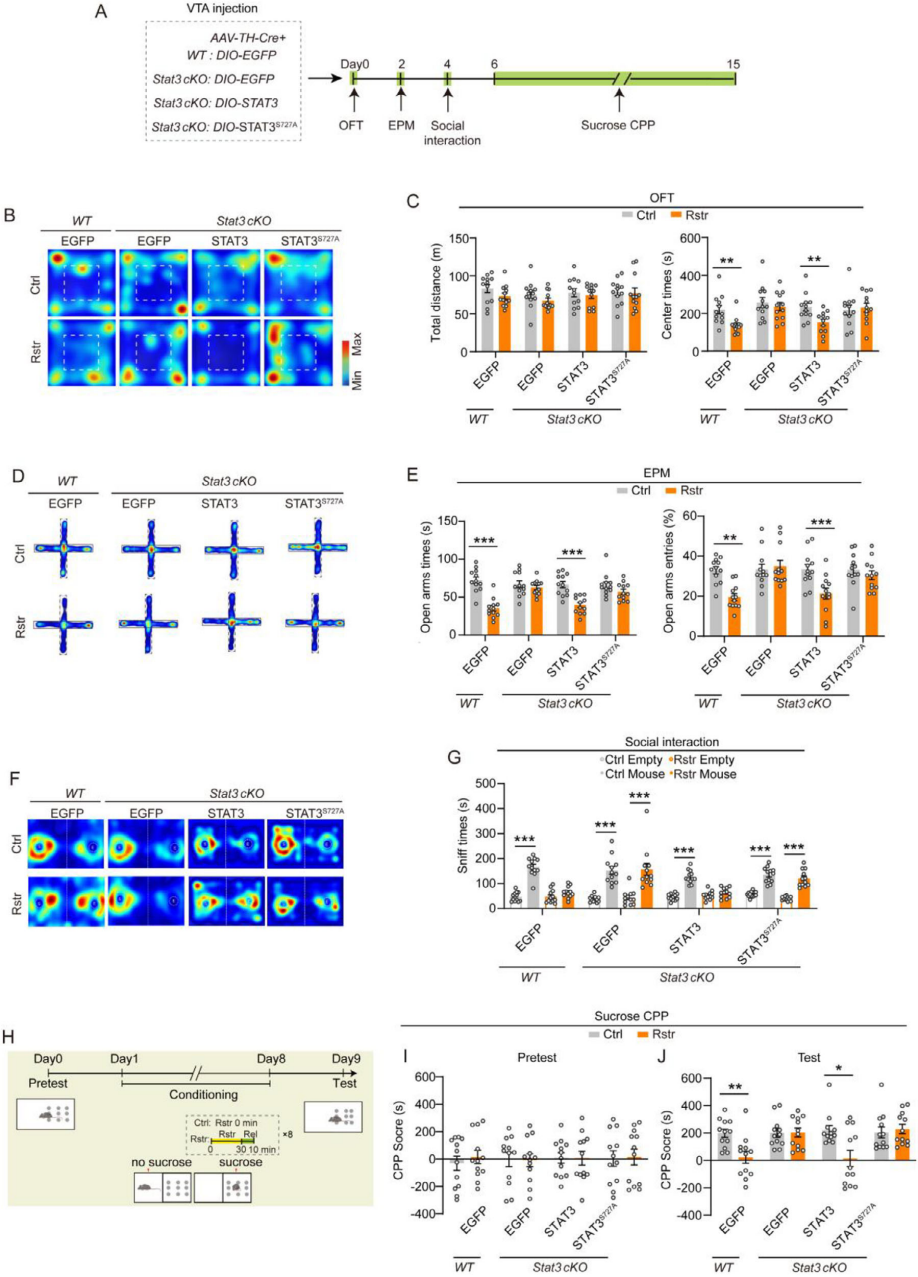
Deficiency of STAT3^Ser727^ phosphorylation in VTA DAergic neurons abolishes anxiety and suppression of reward sensitivity induced by acute restraint stress. A) Schematic diagram illustrating virus injection and behavioral testing in mice with or without 30‐min restraint. *AAV‐TH‐Cre* and Cre‐dependent STAT3 constructs were injected into the VTA of *Stat3^flox+/+^* mice and their WT littermates. B–C) Representative heatmaps of behavior trajectory (B) and quantification of total distance traveled and time spent in the center area during the OFT (C). D–E) Representative heatmaps of behavior trajectory (D) and quantitative (E) of time spent in and entries into the open arms of the EPM test with or without restraint stress. F–G) Social interaction test: Representative heatmaps of behavior trajectory (F) and quantitative (G) of the time exploring a cage containing a novel mouse or an empty cage. H–J) Sucrose CPP. Schematic diagram (H) and CPP scores during the pretest (I) and test (J) sessions. Mice were subjected to 30 min of acute restraint 10 min before each sucrose conditioning session. *n =* 12 mice per group. Data are presented as mean ± SEM. ^*^ *p* < 0.05, ^**^ *p* < 0.01, ^***^ *p* < 0.001; *p*‐values are calculated using Mann‐Whitney U test (C, E, G, I, J), Welch's t‐test (G), or two‐tailed unpaired Student's t‐test (C, E, G, I, J).

The role of STAT3^Ser727^ phosphorylation in stress‐induced behavior changes was further investigated. STAT3, STAT3^S727A^, or EGFP control were overexpressed in the VTA DAergic neurons of *Stat3 cKO* and WT mice (**Figure** **5**A). Behavioral experiments were conducted with or without acute restraint stress (Figure 5A). *Stat3 cKO* mice expressing EGFP did not show elevated anxiety levels in the OFT or EPM after acute restraint stress (Figure 5B–E). In contrast, overexpression of STAT3 in VTA DAergic neurons restored restraint‐induced anxiety in *Stat3 cKO* mice. However, overexpression of the STAT3^S727A^ mutant failed to restore this effect (Figure 5B–E).

In social interaction and sucrose CPP tests, *Stat3 cKO* mice expressing EGFP showed reduced social and sucrose preferences after acute restraint stress (Figure 5F–J). Overexpression of STAT3 in VTA DAergic neurons restored social and sucrose preferences in *Stat3 cKO* mice, reversing the restraint‐induced suppression (Figure 5F–J). However, this effect was absent in *Stat3 cKO* mice overexpressing the STAT3^S727A^ mutant (Figure 5F–J).

These results suggest the sustained suppression of VTA–NAc neuronal activity after acute restraint stress is associated with heightened anxiety and a reduced preference for natural reward. STAT3^Ser727^ phosphorylation in VTA DAergic neurons may serve as a key modulator of these stress‐induced neural and behavioral adaptations.

### 2.6 Upregulation of O‐GlcNAcylation in VTA DAergic Neurons Abolishes Anxiety and the Suppression of Reward‐Seeking Behaviors Induced by Acute Restraint Stress, and this Effect is Impeded by the Phosphomimetic STAT3^S727D^ Mutant

**Figure 6:**
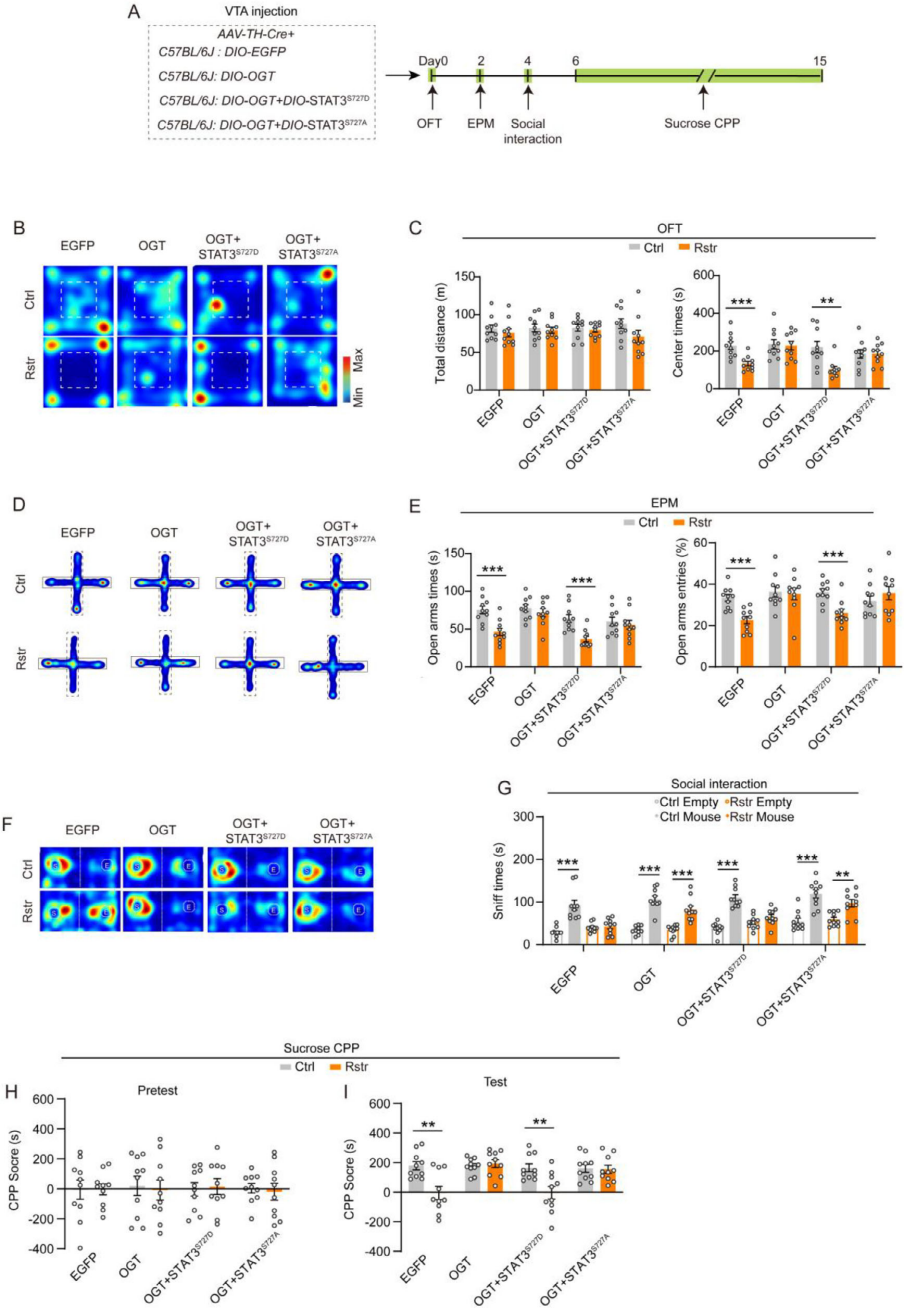
Overexpression of OGT in VTA DAergic neurons abolishes both anxiety and reduced reward‐seeking behaviors induced by acute restraint stress, and this effect is impeded by the phosphomimetic STAT3^S727D^ mutant. A) Schematic diagram depicting virus injection and behavior testing procedures with or without 30‐min restraint. *AAV‐TH‐Cre*, *Cre‐dependent OGT*, and *STAT3* constructs were injected into the VTA of mice. B–C) Representative heatmaps (B) and quantitative analysis (C) of the OFT in mice. D–E) Representative heatmaps (D) and quantitative analysis (E) of the EPM in mice. F–G) Representative heatmaps (F) and quantitative analysis (G) of the time spent exploring a cage containing a novel mouse or an empty cage. H–J) Sucrose CPP test. CPP scores (s) during the pretest and test sessions. *n =* 10 mice per group. Data are presented as mean ± SEM. ^*^ *p* < 0.05, ^**^ *p* < 0.01, ^***^ *p* < 0.001; *p*‐values are calculated using Mann‐Whitney U test (C, E, G), Welch's t‐test (H), or two‐tailed unpaired Student's t‐test (C, E, G, H, I).

O‐GlcNAcylation in VTA DAergic neurons can be upregulated by overexpressing OGT or knocking down OGA (Figure S5, Supporting Information). *AAV‐TH‐Cre* and Cre‐dependent AAVs encoding OGT, STAT3 mutants, or EGFP were injected into the VTA of C57BL/6J mice to investigate the roles of O‐GlcNAcylation and STAT3^Ser727^ phosphorylation in behavioral responses to acute restraint stress (**Figure** **6**A).

In the OFT and EPM tests, EGFP‐expressing mice exhibited significantly elevated anxiety levels after acute restraint stress (Figure 6B–E). In contrast, OGT‐expressing mice did not exhibit increased anxiety levels, suggesting a buffering role of OGT‐mediated O‐GlcNAcylation against restraint‐induced anxiety. Coexpression of the STAT3^S727D^ mutant with OGT in VTA DAergic neurons restored the elevated anxiety levels after acute restraint stress, resembling that observed in the EGFP‐expressing group (Figure 6B–E). However, mice coexpressing the STAT3^S727A^ mutant with OGT did not exhibit elevated anxiety after acute restraint stress (Figure 6B–E).

In social interaction and sucrose CPP tests, EGFP‐expressing mice exhibited reduced social and sucrose preferences after acute restraint stress (Figure 6F–I). In contrast, OGT‐expressing mice maintained their social and sucrose preferences after acute restraint stress, further supporting the buffering role of OGT‐mediated O‐GlcNAcylation. Coexpression of the STAT3^S727D^ mutant with OGT in VTA DAergic neurons led to reductions in social and sucrose preferences, resembling that observed in the EGFP‐expressing group. In contrast, mice coexpressing the STAT3^S727A^ mutant with OGT maintained their social and sucrose preferences after acute restraint stress (Figure 6F–I).

**Figure 7:**
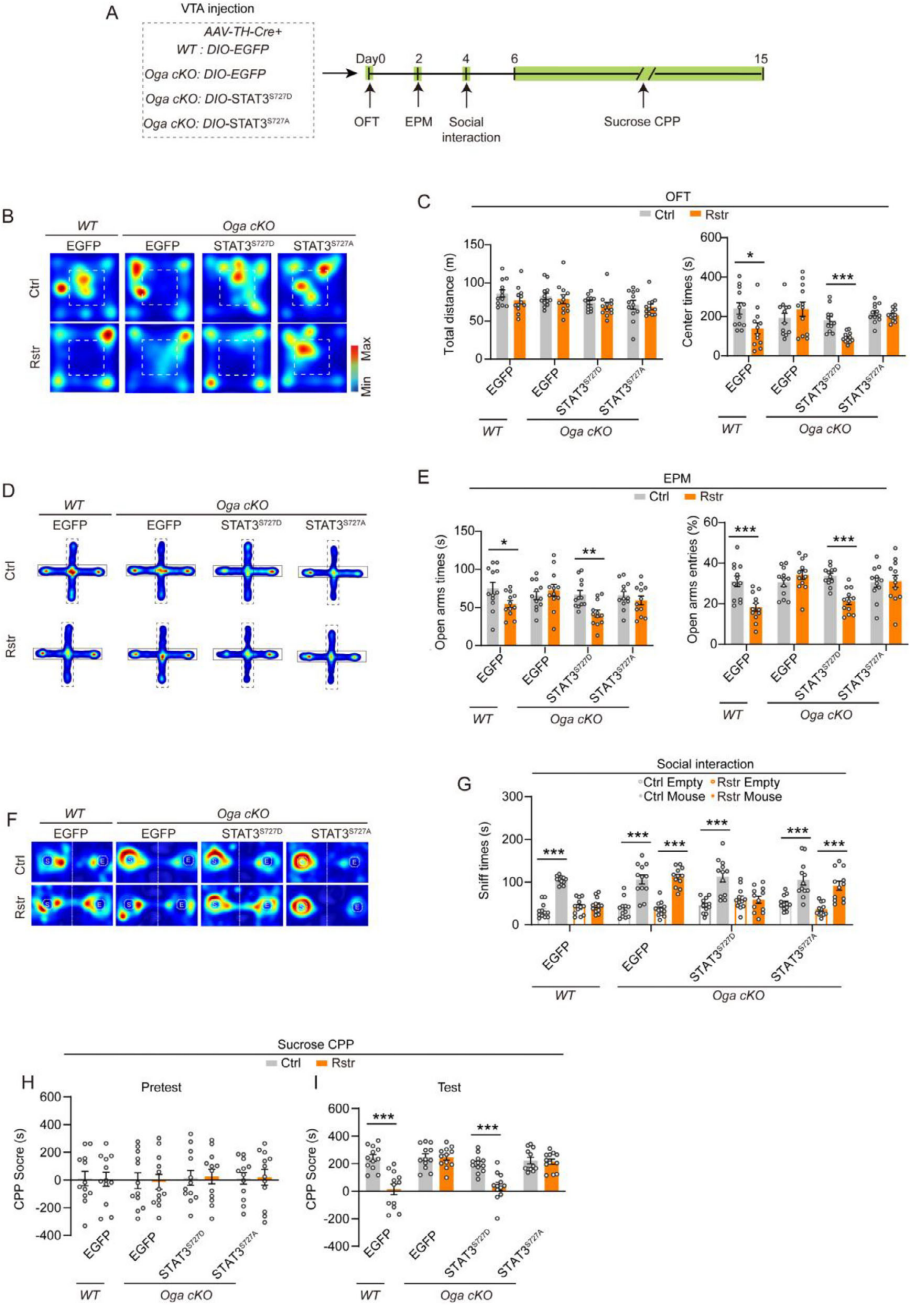
Knockdown of *Oga* in VTA DAergic neurons abolishes both anxiety and reduced reward‐seeking behaviors induced by acute restraint stress, and this effect is impeded by the phosphomimetic STAT3^S727D^ mutant. A) Schematic diagram depicting virus injection and behavioral testing. *AAV‐TH‐Cre*, *Cre‐dependent EGFP, or STAT3* were injected into the VTA of *Oga ^flox+/+^* and *Oga ^flox‐/‐^* mice. B–C) OFT. Representative heatmaps of behavioral trajectory (B) and quantitative analysis of total distance traveled and time spent in the center area (C) in restraint and control groups of mice. D–E) EPM. Representative heatmaps of behavioral trajectory (D) and quantitative analysis of time spent and entries into the open arms (E) in restraint and control groups of mice. F–G) Social interaction tests. Representative heatmaps of behavioral trajectory (F) and quantitative analysis of time spent exploring a cage containing either a novel mouse or an empty cage (G) in restraint and control groups of mice. H–I) Sucrose CPP test: CPP scores during the pretest (H) and test (I) sessions. Mice underwent 30 min of acute restraint 10 min before each sucrose conditioning session. *n =* 12 mice per group. Data are presented as mean ± SEM. ^*^ *p* < 0.05, ^**^ *p* < 0.01, ^***^ *p* < 0.001; *p*‐values are calculated using Mann‐Whitney U test (C, G, I), Welch's t‐test (G), or two‐tailed unpaired Student's t‐test (C, E, G, H, I).

*AAV‐TH‐Cre*, combined with Cre‐dependent AAVs encoding STAT3 mutants or EGFP control, was injected into the VTA of *Oga cKO* mice and their WT littermates (**Figure** **7**A). In the OFT and EPM tests, WT mice expressing EGFP in VTA DAergic neurons exhibited significantly elevated anxiety levels after acute restraint stress. In contrast, *Oga cKO* mice expressing EGFP did not exhibit elevated anxiety levels after acute restraint stress (Figure 7B–E). *Oga cKO* mice overexpressing STAT3^S727D^ in VTA DAergic neurons exhibited elevated anxiety levels after acute restraint stress, comparable to those observed in the WT group. However, *Oga cKO* mice overexpressing STAT3^S727A^ in VTA DAergic neurons did not exhibit elevated anxiety levels after acute restraint stress (Figure 7B–E).

In social interaction and sucrose CPP tests, WT mice expressing EGFP in VTA DAergic neurons exhibited reduced social and sucrose preferences after acute restraint stress. In contrast, *Oga cKO* mice expressing EGFP did not show changes in social or sucrose preferences after acute restraint stress (Figure 7F–I). Acute restraint led to significant reductions in social and sucrose preferences in *Oga cKO* mice overexpressing STAT3^S727D^, comparable to those observed in the WT group. Conversely, social and sucrose preferences in *Oga cKO* mice overexpressing STAT3^S727A^ were unaffected by acute restraint stress (Figure 7F–I).

These results suggest that O‐GlcNAcylation in VTA DAergic neurons may mitigate the increased anxiety and reduced reward sensitivity induced by acute restraint stress. However, STAT3^Ser727^ phosphorylation may counteract the buffering effects of O‐GlcNAcylation against stress.

### 2.7 O‐GlcNAcylation and STAT3^Ser727^ Phosphorylation Mediate the Upregulation of Gamma‐Aminobutyric Acid (GABA) Receptor Expression in VTA DAergic Neurons in Response to Acute Restraint Stress

**Figure 8:**
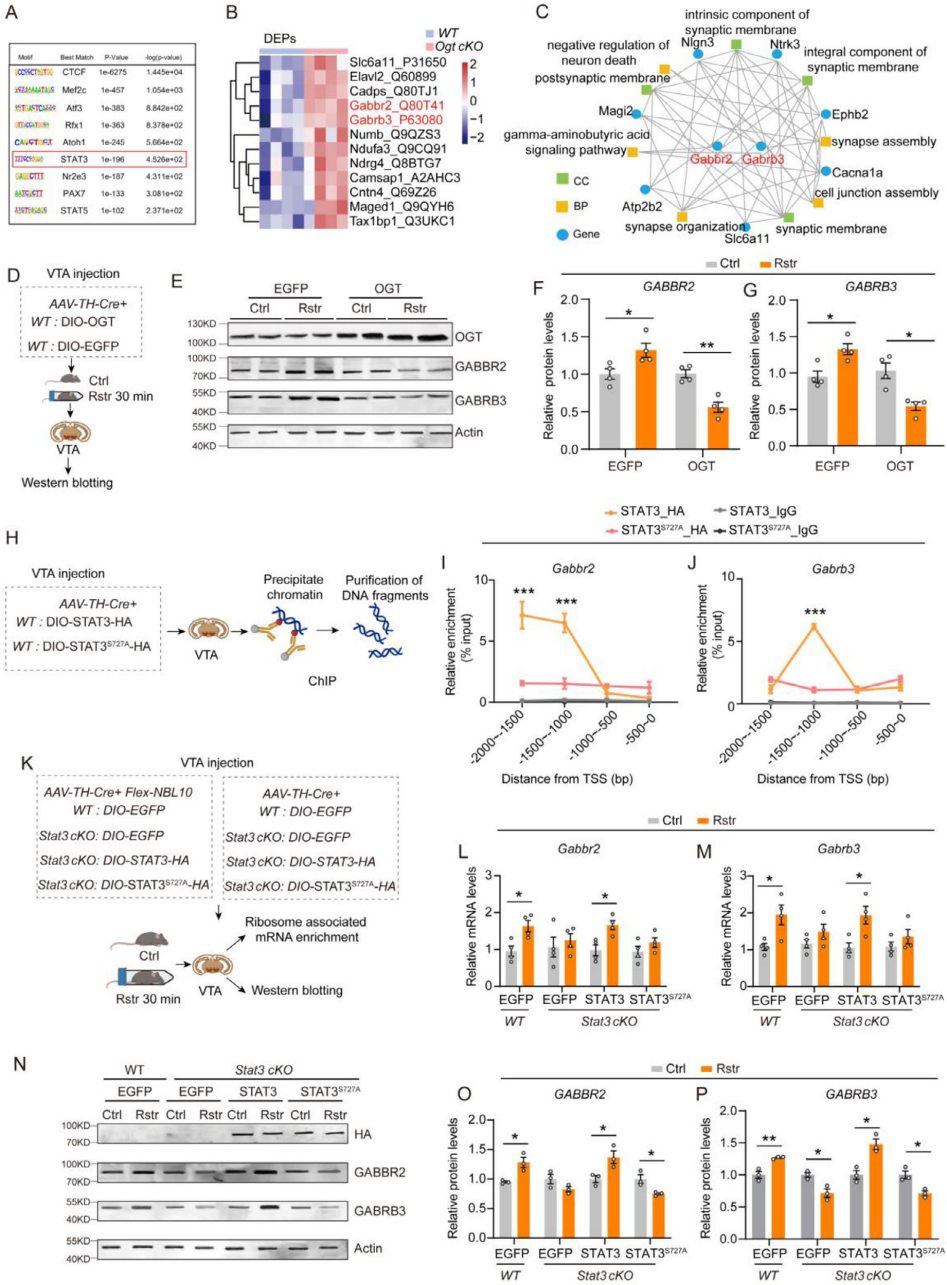
O‐GlcNAcylation and STAT3^Ser727^ phosphorylation mediate acute restraint stress–induced upregulation of GABA receptor expression in VTA DAergic neurons. A) The most enriched transcription factor binding motifs in DARs altered by *Ogt* cKO in the VTA DAergic neurons. B) Heatmap of DEPs in the VTA between the *Ogt ^flox+/Y^* and *Ogt ^flox‐/Y^* group. C) GO enrichment analysis of the co‐expression network between DARs and DEPs. D) Schematic diagram of the western blot experiment. AAV‐TH‐Cre and DIO‐EGFP or DIO‐OGT were co‐injected into the VTA of WT mice. VTA tissues were collected 30 min after acute restraint stress. E–G) Representative immunoblots (E) and quantification of GABBR2 (F) and GABRB3 (G) protein levels in the VTA, normalized to actin. *n =* 4 samples from 8 mice per group. H) Schematic diagram of STAT3‐specific ChIP assay in VTA tissue. *AAV‐TH‐Cre* and Cre‐dependent constructs encoding HA‐tagged STAT3 or STAT3^Ser727A^ were injected into the VTA of mice. I–J) qPCR analysis of STAT3 enrichment at genomic regions ranging from −2000 to −1000 bp upstream of the *Gabbr2* TSS, and from −1500 to −1000 bp upstream of the *Gabbr3* TSS, as assessed by HA‐specific ChIP. Rabbit IgG was used as a negative control. Enrichment levels were normalized to input. *n =* 4 samples from 12 mice per group. K) Schematic diagram illustrating the detection of *Gabbr2* and *Gabrb3* expression in VTA DAergic neurons. For transcript‐level analysis, *AAV‐TH‐Cre, Flex‐NBL10*, and Cre‐dependent constructs encoding EGFP, STAT3, or STAT3^S727A^ were injected into the VTA of *Stat3^flox+/+^* or *Stat3^flox‐/‐^* mice. For protein‐level analysis, the same constructs (excluding Flex‐NBL10) were used. L–M) qRT‐PCR analysis of *Gabbr2* and *Gabbr3* transcripts in VTA DAergic neurons from mice with or without acute restraint stress. *n =* 4 samples from 8 mice per group. N–P) Representative immunoblots (N) and quantification of GABBR2 (O) and GABRB3 (P) protein levels in the VTA, normalized to actin. *n =* 3 samples from 6 mice per group. Data are presented as mean ± SEM.^*^ *p* < 0.05, ^**^ *p* < 0.01, ^***^ *p* < 0.001; *p*‐values are calculated using Welch's t‐test (M), two‐tailed unpaired Student's t‐test (L, M, O, P), Two‐way RM ANOVA followed by *Bonferroni post hoc* test (I, J), Two‐way ANOVA (F) or Scheirer‐Ray‐Hare Test (G) followed by *Bonferroni post hoc* test.

Motif prediction analysis of DARs in VTA DAergic neurons from ATAC‐seq data identified STAT3 as a potential transcription factor regulated by OGT (**Figure** **8**A). Analysis of DEPs in VTA tissue further revealed that *Ogt cKO* significantly upregulated genes encoding the GABA receptor subtypes *Gabbr2* and *Gabrb3* (Figure 8B). Moreover, GO enrichment analysis of the co‐expression network constructed from DARs and DEPs indicated that *Gabbr2* and *Gabrb3* occupy central positions within the network (Figure 8C). To assess the role of O‐GlcNAcylation in regulating GABA receptor expression under stress, OGT was overexpressed in VTA DAergic neurons of WT mice, with EGFP as a control (Figure 8D, Figure S10A, Supporting Information). After acute restraint, GABBR2 and GABRB3 protein levels increased in the EGFP group but not in the OGT‐overexpressing group (Figure 8E,F). A similar pattern was observed in *Oga cKO* mice, which did not show stress‐induced upregulation of these proteins, unlike their WT littermates (Figure S10B–F, Supporting Information). These findings suggest that elevated O‐GlcNAcylation may suppress stress‐induced GABA receptor expression and modulate inhibitory signaling in VTA DAergic neurons.

To investigate how STAT3 regulates the transcription of *Gabbr2* and *Gabrb3*, AAV‐TH‐Cre and Cre‐dependent AAVs encoding HA‐tagged STAT3 or its phosphorylation‐deficient mutant STAT3^S727A^ were delivered into the VTA of WT mice. Chromatin immunoprecipitation (ChIP) assays were performed to analyze the DNA binding of STAT3 and STAT3^S727A^ using an anti‐HA antibody (Figure 8H). HA‐ChIP qPCR results showed that STAT3 was enriched in regions ranging from −2000 to −1000 bp upstream of the TSS of *Gabbr2* and from −1500 to −1000 bp upstream of the TSS of *Gabrb3*, whereas STAT3^S727A^ was not significantly enriched in these regions (Figure 8I,J).

mRNA was isolated from *Stat3 cKO* and WT mice expressing either various STAT3 constructs or EGFP controls in VTA DAergic neurons (Figure S10G, Supporting Information). qRT‐PCR analysis revealed that *Gabbr2* and *Gabrb3* mRNA expression in VTA tissues was significantly upregulated after 30 min of acute restraint stress in WT littermates, whereas no significant changes were observed in the *Stat3 cKO* mice expressing EGFP (Figure S10H,I, Supporting Information). Restraint‐induced upregulation of *Gabbr2* and *Gabrb3* was restored in the VTA of *Stat3 cKO* mice overexpressing STAT3, whereas *Stat3 cKO* mice overexpressing STAT3^S727A^ did not exhibit this upregulation (Figure S10H,I, Supporting Information).

Furthermore, ribosome‐associated mRNA from DAergic neurons was enriched from VTA tissues of *Stat3 cKO* and WT mice (Figure 8K). Consistently, expression of *Gabbr2* and *Gabrb3* was significantly increased in WT DAergic neurons after acute restraint, whereas no significant changes were observed in *Stat3 cKO* neurons expressing EGFP (Figure 8L,M). Restraint‐induced upregulation of *Gabbr2* and *Gabrb3* was restored in the VTA DAergic neurons of *Stat3 cKO* mice overexpressing STAT3, whereas this upregulation was absent in *Stat3 cKO* mice overexpressing STAT3^S727A^ (Figure 8L,M).

Additionally, GABBR2 and GABRB3 protein levels were examined in VTA tissues from *Stat3 cKO* and WT mice (Figure 8N). Consistent with the transcriptional results, protein expression of GABBR2 and GABRB3 was significantly increased in WT mice after acute restraint, but not in *Stat3 cKO* mice expressing EGFP (Figure 8O,P). The stress‐induced upregulation of GABBR2 and GABRB3 was restored in *Stat3 cKO* mice by overexpressing STAT3, whereas it was decreased in the STAT3^S727A^ group.

**Figure 9:**
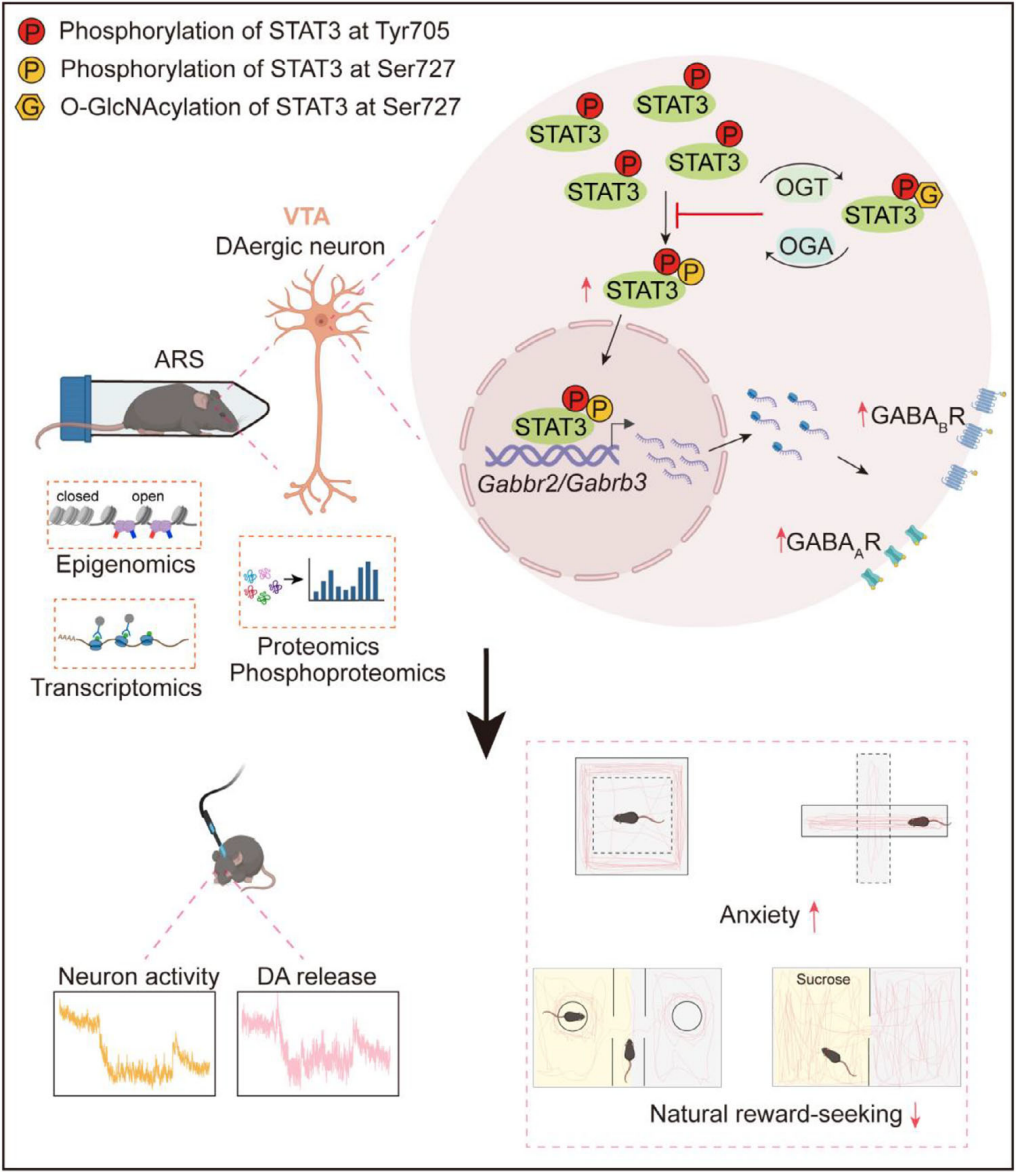
Working model: O‐GlcNAcylation and STAT3^Ser727^ phosphorylation in VTA DAergic neurons mediate behavioral adaptations to acute stress. Acute restraint stress enhances the phosphorylation of STAT3 at Ser727 in DAergic neurons in mice. This, in turn, mediates the upregulation of GABBR2 and GABRB3, a decrease in neuronal activity, and the promotion of anxiety‐like behavior and reduced reward‐seeking after restraint. Meanwhile, O‐GlcNAcylation is upregulated in the VTA to counteract STAT3 phosphorylation, thereby preventing stress‐induced neuronal and behavioral changes and maintaining emotional homeostasis.

In summary, the present study demonstrates that acute restraint stress enhances the phosphorylation of STAT3 at Ser727 in the VTA, which is required for the upregulation of GABA receptor subunits GABBR2 and GABRB3, the suppression of neuronal activity, and the development of anxiety‐like behavior and reduced reward seeking in mice. Meanwhile, O‐GlcNAcylation in the VTA increased as a potential stress‐buffering mechanism, antagonizing STAT3 phosphorylation and thereby preventing stress‐induced neuronal and behavioral adaptations (**Figure** **9**). These findings highlight the critical role of dynamic PTMs in the mesolimbic DA system in response to acute stress, ensuring the maintenance of neuronal and emotional homeostasis.

## 3 Discussion

DA is released by DAergic neurons in the VTA into key brain regions, including the NAc^[^ ^39^ ^]^ and the amygdala.^[^ ^40^ ^]^ This release of DA regulates behavior,^[^ ^41^ ^]^ motivation,^[^ ^42^ ^]^ and emotional states.^[^ ^43^ ^]^ Acute stress, often triggered by environmental or psychological challenges, disrupts the balance of DA release,^[^ ^13^, ^44^, ^45^ ^]^ and is associated with heightened anxiety, reduced reward‐seeking behavior, and increased susceptibility to mood disorders such as anxiety and depression.^[^ ^2^, ^46^ ^]^ Our study uncovered a dynamic interplay between O‐GlcNAcylation and STAT3 phosphorylation in VTA DAergic neurons, which regulates neuronal activity and behavioral adaptations in response to acute restraint stress. These findings elucidate how counterbalancing PTMs can modulate transcription factors, thereby influencing gene transcription, neuronal activity, and behavior changes in response to acute restraint stress. This process contributes to the maintenance of emotional stability and may provide a molecular basis for neuronal adaptation to acute stress stimuli.

The canonical activation pathway of STAT3 relies on the phosphorylation of tyrosine 705 in response to cytokines, growth factors, and pathological conditions such as oncogene activation.^[^ ^47^ ^]^ Although phosphorylation of tyrosine 705 (p‐Y705) is the primary PTM described for STAT3, phosphorylation at serine 727 (p‐S727) is increasingly recognized as a functionally important PTM that regulates STAT3 activity in a non‐canonical manner. Recent studies have shown that STAT3 can exert its pro‐tumoral effects through p‐S727 in a manner independent of p‐Y705,^[^ ^48^, ^49^ ^]^ and that this modification is required for the expression of a gene subset distinct from those regulated by pY705.^[^ ^50^ ^]^

The present findings demonstrate that both p‐S727 and p‐Y705 of STAT3 were increased by acute restraint stress. Elevated levels of O‐GlcNAcylation in VTA DAergic neurons counteracted the increase in p‐S727, but not p‐Y705, of STAT3 induced by acute restraint stress. This modulation may help preserve DAergic activity and restore reward‐seeking behavior after acute restraint stress. These findings suggest that O‐GlcNAcylation buffers the negative effects of acute restraint stress and may contribute to stress resilience. The modulation of neuronal activity by O‐GlcNAcylation may function as a molecular feedback mechanism that compensates for the inhibitory effects of STAT3^Ser727^phosphorylation. Ultimately, this feedback may help preserve emotional homeostasis under stressful conditions.

GABA, the brain's primary inhibitory neurotransmitter, plays a crucial role in modulating neuronal excitability.^[^ ^51^, ^52^ ^]^ In the VTA, GABAergic signaling regulates DAergic activity, ensuring the proper functioning of DAergic neurons and preventing excessive excitability.^[^ ^53^, ^54^ ^]^ In this study, the GABA receptor subunits GABBR2 and GABRB3 were identified as potential downstream targets of STAT3, and their expression may be regulated by the molecular interplay between O‐GlcNAcylation and phosphorylation. Although alterations in GABBR2 and GABRB3 expression were observed at both the mRNA and protein levels, these findings are insufficient to clarify their specific roles in VTA function or behavioral regulation in response to acute stress. Moreover, elevated O‐GlcNAcylation is associated with reduced expression of these GABA receptor subunits. While such molecular changes may influence DAergic neuronal activity and contribute to maintaining DA release within an adaptive range under acute stress, their exact physiological relevance remains to be determined. Further studies, including electrophysiological recordings, are needed to establish whether these molecular changes directly impact dopaminergic regulation and behavioral outcomes.

While previous studies on the brain's adaptive response to acute stress have primarily focused on neural circuit‐level processes, such as synaptic plasticity^[^ ^55^, ^56^ ^]^ and feedback loop regulation within the DAergic system,^[^ ^57^, ^58^ ^]^ our study highlights the pivotal role of PTMs in modulating gene expression and transcriptional responses. O‐GlcNAcylation, a dynamic PTM involving the addition of N‐acetylglucosamine (GlcNAc) to serine or threonine residues of proteins,^[^ ^25^ ^]^ plays a key role in stress adaptation by influencing not only neuronal activity but also broader transcriptional programs governing stress adaptation.

The antagonistic interaction between O‐GlcNAcylation and STAT3 phosphorylation may represent a regulatory mechanism contributing to the brain's adaptation to acute restraint stress. Through its modulation of GABA receptor expression and STAT3 phosphorylation, O‐GlcNAcylation may fine‐tune DAergic neuronal activity, thereby supporting emotional regulation and promoting recovery from acute stress.

Given the pivotal role of DAergic signaling in emotional regulation,^[^ ^59^ ^]^ our findings may provide a rationale for exploring pharmacological strategies targeting O‐GlcNAcylation or STAT3 phosphorylation to buffer DAergic function against stress‐induced impairments and promote emotional stability.

However, several important questions remain unresolved. While this study focused on the acute restraint stress model, future investigations are needed to determine how the interplay between O‐GlcNAcylation and STAT3 phosphorylation functions under chronic stress conditions. In addition to the identified GABA receptor subunits as key downstream targets of STAT3 in response to stress, other genes and proteins —particularly those related to neuronal plasticity, synaptic function, and cellular resilience—may also play important roles in this regulatory network.

In conclusion, our study provides novel insights into the molecular mechanisms of stress adaptation, highlighting the antagonistic interaction between O‐GlcNAcylation and STAT3 phosphorylation in VTA DAergic neurons. This molecular pathway may be critical for regulating neuronal and behavioral adaptations to acute restraint stress. Further research is required to elucidate its involvement in behavioral changes induced by chronic stress, as well as its potential as a therapeutic target in stress‐related psychiatric disorders.

## 4 Experimental Section

### Animals

Male *C57BL/6J* mice, aged 6‐8 weeks, were purchased from the Shanghai Laboratory Animal Center (CAS, Shanghai, China). CRISPR‐Cas9 mediated construction of *Stat3^flox^* cKO mice (NO. NM‐CKO‐200050) targeting exons 12–14 was generated by GenoBioTX (Shanghai, China). CRISPR‐Cas9 mediated construction of *Oga^flox^* cKO mice (NO. S‐CKO‐16385) targeting exons 2‐4 was generated by Cyagen (Suzhou, China). *Ogt^flox^* mice (No. 004860) were purchased from The Jackson Laboratory (CA, USA). Mice were bred to C57BL/6J mice for more than six generations and were group‐housed and maintained on a 12 h light‐dark cycle (light on from 8 a.m. to 8 p.m.) with access to food and water available ad libitum. Behavioral experiments were conducted using male mice aged 8–12 weeks. All experiments were performed following the National Institutes of Health Guide for the Care and Use of Laboratory Animals and approved by the Animal Care and Use Committee of Fudan University (No. 20220228‐030).

### Virus Constructs

The coding sequence of mouse *Stat3*, obtained from the *pLEGFP‐WT‐STAT3* vector (Addgene #71450), was subcloned into the *pAAV‐EF1a‐DIO‐EYFP* vector (Addgene #27056) to generate *pAAV‐EF1a‐DIO‐STAT3‐HA*. *pAAV‐EF1a‐DIO‐STAT3^S727D^‐HA* and *pAAV‐EF1a‐DIO‐STAT3^S727A^‐HA* were generated by point mutations from the *pLEGFP‐WT‐STAT3* vector and subcloned into the *pAAV‐EF1a‐DIO‐EYFP* vector. *pAAV‐CMV‐DIO‐OGT‐HA* was generated as described previously.^[^ ^60^ ^]^ These AAV vectors were packaged into serotype 9 by Obio Technology Co., Ltd (Shanghai, China). *AAV_9_‐EF1a‐FLEX‐NBL10‐HA* (Addgene #69971) was packaged by Taitool Bioscience Co., Ltd. (Shanghai, China). *AAV_9_‐EFla‐DIO‐mCherry, AAV_9_‐EF1a‐DIO‐EGFP*, and *AAV_9_‐TH‐Cre* were purchased from Obio Technology Co., Ltd (Shanghai, China). *AAV_9_‐CAG‐FLEX‐jGCaMP7b*, *AAV_9_‐hSyn‐DA4.4*, and *AAV_9_‐EF1a‐DIO‐H2B‐EGFP* were purchased from BrainVTA Co., Ltd (Wuhan, Hubei, China).

### Stereotaxic Surgery

Mice were anesthetized with 2% isoflurane and positioned in a stereotactic instrument (Stoelting, Kiel, WI, USA) for surgery. Virus microinjections were administered using 33‐gauge needles attached to a 10‐µL microsyringe (Hamilton, Bonaduz, Switzerland). The stereotaxic coordinates targeted for the VTA were −3.1 mm AP; ±0.9 mm ML (angled 10° laterally from the midline); −4.4 mm DV. The coordinates for the nucleus accumbens (NAc) were +1.4 mm AP; ±1.4 mm ML (also angled 10° laterally from the midline); −4.4 mm DV. Each site received a 0.3 µL injection of purified and mixed AAV (10^12^ IU/mL) at a slow rate of 0.1 µL/min. 200 µm ‐ diameter 0.37 NA optic‐fiber cannulas (Inper Tech, Hangzhou, Zhejiang, China) were implanted above the VTA at −4.30 mm DV or the NAc at −4.30 mm DV. All mice were allowed at least 3 weeks of recovery before subsequent experiments.

### Open‐Field Test

A locomotor activity‐monitoring system (43.2 cm × 43.2 cm × 30.5 cm, Med‐Associates, USA) was used to assess the mice's movement. After 30 min of acute restraint stress, the mice were returned to their cages and allowed to rest for 10 min before testing. Each mouse was placed in the center of the open field and allowed to explore freely for 30 min. The center zone was defined as a square encompassing 50% of the open field area. The time spent in the center zone and the total distance traveled were recorded and analyzed.

### Elevated‐Plus‐Maze Test

The elevated plus maze consisted of four arms (34.5 cm length × 6.3 cm width × 19.5 cm height) and a central platform positioned 75 cm above the floor. Two arms were enclosed with 20 cm high dark walls (closed arms), while the other two had 0.8 cm high ledges (open arms). The four arms were perpendicular to each other. After 30 min of acute restraint stress, the mice were returned to their cages and allowed to rest for 10 min before testing. Mice were placed in the center and allowed to explore the maze freely for 6 min. Their behavior was recorded by a camera positioned above the maze and analyzed using the EthoVision XT 8.5 video‐tracking program (Leesburg, VA, USA). The maze was cleaned with 75% ethanol before each trial to eliminate olfactory cues.

### Social Interaction Test

As previously described,^[^ ^61^ ^]^ mice with or without 30 min of acute restraint stress were placed in the center of a three‐chambered arena (60 × 40 × 22 cm) for a 10‐min habituation period. Two round‐wire cages were placed in the lateral chambers during the test period. One cage was empty, while the other contained an unfamiliar mouse (previously habituated in the cage for three days, 10 min per day). These cages allowed nose contact through the bars but prevented extensive physical interaction. The unfamiliar mouse was changed every two trials, and its position alternated between cages to avoid place preference. The cages were cleaned with 75% ethanol and dried between trials. Each session was recorded on video, and the time spent by the test mouse exploring (approaching, sniffing, or rearing within 2 cm of the cage) the stranger mouse and the empty cage was measured.

### Sucrose Conditioned Place Preference

Sucrose CPP was conducted using a two‐chamber apparatus (15 × 15 × 20 cm) with distinct visual and tactile environments (Med Associates, St. Albans, VT, USA) as previously reported.^[^ ^62^ ^]^ The procedure included three sessions: pre‐test, conditioning, and test. During the pre‐test session (day 1), mice were placed in the center of the apparatus and allowed to explore both chambers freely for 20 min. The time spent in each chamber was recorded, and mice that strongly preferred one chamber (>13 min) were excluded from the study. During the conditioning sessions (days 2–9), the mice were returned to their cages and allowed to rest for 10 min following 30 min of acute restraint stress. Then, the mice were confined to one chamber, where they were paired with surose pellets for 30 min. 6 h later, they were confined to the other empty chamber for another 30 min. During the testing session (day 10), mice were placed in the center of the apparatus and allowed to freely explore both chambers for 20 min. The time spent in each chamber was recorded. The CPP score was calculated as the time (in seconds) spent in the chamber with sugar pellets minus the time spent in the empty chamber.

### Fiber Photometry Recordings

The 470 nm laser power at the tip of the optical fiber was adjusted to 10–20 µW to minimize bleaching. The sampling frequency of the GCaMP or genetically encoded DA sensor (DA4.4) signals was set at 100 Hz using a fiber photometry system (Thinker Tech Nanjing Biotech, Nanjing, China). Recordings were conducted during behavioral tasks including sucrose licking, social sniffing of a male stranger, and acute restraint stress.

For sucrose water‐licking, mice were water‐deprived for 12 h before the test. Water delivery was controlled by a microcontroller‐based behavioral system running on Tracking Master V3.0 software (VanBi Intelligent Technology Co., Ltd., Shanghai, China), and the licking was detected by a custom‐made lickometer equipped with a capacitive touch sensor and microcontroller. Upon each lick, 20 µL of water was dispensed, followed by a 10‐s timeout. The onset of licking triggered a TTL signal, synchronously recorded with fluorescence signals, and the fluorescence signaling during the first five licking events was analyzed. For social interaction, the mice were habituated to a rectangular open field (40 × 40 × 40 cm), and a juvenile (5‐6 weeks old) mouse was introduced as an intruder. Social behaviors and fluorescence signals were recorded simultaneously. “Sniffing” was defined as the male mouse's nose approaching the face or body of the stranger.

For acute restraint, mice were exposed to the habituated open field for 5 min, followed by placement in a restraining tube for 10 min, and then released for an additional 5 min.

Both mouse behavior and fluorescence signals were recorded simultaneously. The data were segmented based on behavioral events within individual trials. The 2 s before sucrose water licking and sniffing a stranger male mouse, and the values 5 min before restraint were used as the baseline respectively. The 470 nm and 410 nm signals were collected separately and normalized to baseline signals. GCaMP signals were analyzed and plotted with MATLAB R2020a (MathWorks, Natick, MA, USA) as previously described.^[^ ^61^ ^]^ ΔF/F = (F–F0)/F where F0 was the mean value of the baseline fluorescence signal and F was the observed fluorescence at a given time point. AUC was calculated by integrating the fluorescence signal from the start of the event to the end, often using the trapezoidal rule. To account for inter‐subject and inter‐experimental variability, fluorescence traces were further normalized using Z‐score transformation: Z = (F – μ) / σ, where μ was the mean and σ was the standard deviation of the baseline period.

### Immunofluorescence

Mice were transcardially perfused with 0.9% saline followed by 4% PFA (prepared in 0.1 m Na2HPO4/NaH2PO4 buffer, pH 7.5). The brains were post‐fixed in 4% PFA at 4 °C for 4 h and then transferred to 30% sucrose/PBS for 3 days. Brains were sliced into 30 µm coronal sections using a Leica CM3050 S Cryostat (Buffalo Grove, IL, USA). For immunostaining, slices were washed in PBS and incubated with primary antibodies overnight at 4 °C. After being washed in PBS, the slices were incubated with secondary antibodies for 2 h and then stained with DAPI (D9534, Sigma–Aldrich, St. Louis, MO, USA) at room temperature. Finally, slices were mounted with an anti‐quenching mounting medium (F4680 Thermo Fisher Scientific, Waltham, MA, USA). The primary antibodies used: anti‐TH (1:1,000, AB152, Sigma–Aldrich); anti‐TH (1:1,000, MAB318, Sigma–Aldrich); anti‐HA (1:500, H6908, Sigma–Aldrich); anti‐O‐GlcNAc (1:200, MA1‐072, Thermo Fisher Scientific). The secondary antibodies used were: Alexa488, Cy3, or Alexa647 (mouse/rabbit; 1:500, Jackson ImmunoResearch, Bar Harbor, ME, USA).

### Co‐Immunoprecipitation

VTA brain tissues were rapidly extracted and homogenized in radioimmunoprecipitation assay (RIPA) buffer containing protease and phosphatase inhibitors. The protein lysate was incubated overnight at 4 °C with an anti‐STAT3 antibody (12640S, CST, 1:100) or anti‐IgG (5415S, CST, 1:100). Pierce Protein A/G magnetic beads (88803, Thermo Scientific) were then added, and the mixture was rotated at 4 °C overnight. After washing the beads, the immunoprecipitated protein complexes were resolved by a 4‐20% BeyoGel™ Plus PAGE (P0468S, Beyotime, Shanghai, China) and subjected to anti‐STAT3 and anti‐O‐GlcNAc immunoblotting.

### Western Blot Analysis

VTA tissues were homogenized and lysed in RIPA Lysis Buffer (P0013B, Beyotime) containing protease inhibitor cocktail (539134, Millipore, MA, USA), Na3VO4 (450243, Sigma–Aldrich), and PMSF (ST506, Beyotime). After centrifugation at 4 °C at 15,000 × g for 30 min, the supernatant protein was quantified with the BCA protein assay kit (23225, Thermo Fisher Scientific) and subsequently applied for Western blot analysis. Protein samples (20 µg) were separated by 4‐20% BeyoGel™ Plus PAGE (P0468S, Beyotime) and transferred to a nitrocellulose membrane (0.45 µm, A29603478, GE Whatman, Maidstone, UK). The membranes were blocked with 5% BSA and incubated with primary antibodies against O‐GlcNAc (1:500, MA1‐072, Thermo Fisher Scientific), STAT3 (1:1000, #12640, CST), phospho‐STAT3^Tyr705^ (1:1000, #9134, CST), phospho‐STAT3^Ser727^ (1:1000, MA5‐32089, Thermo Fisher Scientific), GABBR2 (1:1000, MA5‐42479, Thermo Fisher Scientific), GABRB3 (1:1000, ab98968, Abcam), Actin (1:2000, A2066, Sigma–Aldrich) overnight at 4 °C, and then incubated with appropriate secondary antibodies at room temperature for 1 h. Blots were imaged on the Odyssey (LI‐COR Biosciences, Lincoln, NE, USA), and the grayscale of each band was analyzed by open‐source software Image J.

### Serum Corticosterone Measurement

To evaluate the endocrine response to acute restraint stress, serum corticosterone levels were quantified using the QuicKey Pro Mouse CORT ELISA Kit (Cat. No. E‐OSEL‐M0001, Elabscience, Wuhan, China) according to the manufacturer's instructions. Briefly, retro‐orbital blood collections were performed on anaesthetized mice immediately after acute restraint or release. The samples were then allowed to clot at room temperature and centrifuged at 3,000 × g for 15 min at 4 °C to obtain serum. 50 µL of serum or standard solution and 50 µL of HRP‐conjugated antibody were added to each well and incubated at 37 °C for 60 min. After five washes, 90 µL of substrate reagent was added and incubated for 15 min at 37 °C. Then, 50 µL of stop solution was added. Absorbance was read at 450 nm, and corticosterone concentrations were calculated from the standard curve.

### Flow Cytometry

Male *Ogt^flox+/Y^* mice (6–8 weeks old) and their littermate controls (*Ogt^flox‐/Y^*) were used. A mix of *AAV‐TH‐Cre* and *AAV‐EF1a‐DIO‐H2B‐EGFP* (1:1 ratio, diluted to 10^12^ IU/ml) was microinjected into the VTA. After a 4‐week recovery, brain tissue containing VTA was dissected using a stereotaxic mold and placed in pre‐chilled 1×HBSS solution (14025092, Thermo Fisher Scientific). The tissue was homogenized with sterilized steel beads. After centrifugation, the pellet was resuspended, mixed with Lysis buffer and Sucrose solution, and centrifuged again. The final pellet was resuspended in 1×HBSS with BSA (B14, Thermo Fisher Scientific) and DAPI. EGFP‐labeled neuron nuclei were then sorted using a flow cytometer (Beckman Coulter, Brea, CA, USA).

Before sorting, a preliminary experiment should optimize the conditions and establish the sorting gate. The flow rate should be adjusted to maintain 100–300 nuclei per second. During sorting, a sample tube with 1×HBSS should be used. Select “0” for continuous sorting, and “Single Cell” mode, and do not display the excluded cell count. Finally, resuspend the sorted nuclei in pre‐chilled 1×HBSS solution.

### In Situ Proximity Ligation Assay

The Duolink PLA kit (DUO92101, Sigma–Aldrich) was used following standard protocols. 20‐µm‐thick slices were incubated with Duolink^®^ blocking solution in a heated humidity chamber for 60 min at 37 °C. The primary antibodies (anti‐OGT, rabbit, 1:100, Thermo Fisher Scientific, PA5‐22071; anti‐STAT3, mouse,1:100, Thermo Fisher Scientific, MA1‐13042; anti‐p‐STAT3^Tyr705^, mouse, 1:100, Cell Signaling Technology, 9145S; anti‐p‐STAT3^Ser727^, mouse, 1:100, Thermo Fisher Scientific, MA5‐15208) were diluted in Duolink® Antibody Diluent and incubated with the slices at 37 °C for 2 h. After washing with buffer A (DUO82049, Sigma–Aldrich), the brain sections were incubated with PLA probes (1:5 dilution) for 1 h at 37 °C. For assessing the protein level of STAT3, phospho‐STAT3^Tyr705^, and phospho‐STAT3^Ser727^, anti‐mouse PLUS PLA probes (DUO92001, Sigma–Aldrich) and MINUS PLA probes (DUO92004, Sigma–Aldrich) were used. For assessing the interaction between STAT3 and OGA/OGT, anti‐rabbit PLUS PLA probes (DUO92002, Sigma–Aldrich) and anti‐mouse MINUS PLA probes (DUO92004, Sigma–Aldrich) were used. Ligation and amplification solutions were prepared and incubated with the slices at 37 °C for 30 min and 100 min sequentially. Slides were washed, stained with DAPI, and mounted with coverslips.

### Confocal Microscopy and Image Analysis

Fluorescence images were acquired using a Nikon A1 confocal microscope (Tokyo, Japan) with a 10 × or 20 × objective lens. Cell counts were performed using ImageJ, with brain regions defined according to the Allen Mouse Brain Reference Atlas. Cell counting and intensity analysis were performed manually by an experimenter who was blinded to the group assignment. The number of TH^+^O‐GlcNAc^+^ cells in the VTA was counted from four slices per mouse. The number and fluorescent intensity of PLA puncta within TH^+^ cells were analyzed using custom MATLAB software as previously described.^[^ ^63^ ^]^

### Protein‐Protein Docking Analysis

As previously described,^[^ ^64^ ^]^ protein structures were retrieved from UniProt using OGT and STAT3 as keywords. The full‐length AlphaFold‐predicted structure of OGT_MOUSE (UniProt ID: Q8CGY8) was selected as the ligand, and the full‐length AlphaFold‐predicted structure of STAT3_MOUSE (UniProt ID: P42227) was chosen as the receptor. Protein‐protein docking was performed using the HDOCKlite v1.1 local server,^[^ ^65^ ^]^ and the protein binding process was visualized in Surface representation.

### Ribosome Affinity Purification and RNA‐Seq

Purification procedures for enriching ribosome‐associated transcripts were performed as previously described.^[^ ^66^ ^]^ Brain tissues containing VTA were isolated and homogenized in a supplemented hybridization buffer containing dithiothreitol, cycloheximide (66‐81‐9, Cayman, MI, USA), heparin (H3393, Sigma–Aldrich), protease inhibitors (04693159001, Roche, Basel, Switzerland) and RNase inhibitor (N2112S, Promega Corporation, Madison, WI, USA). The supernatant was incubated with an anti‐HA antibody (H6908; Sigma–Aldrich) and Dynabeads Protein G (10003D, Thermo Fisher Scientific) and rotated at 4 °C overnight. After washing with high‐salt wash buffer, RNA was isolated from the Dynabeads using TRIzol LS (15596026, Thermo Fisher Scientific). The quality of the purified mRNA was evaluated using an Agilent RNA 6000 Pico Kit (5067‐1513, Agilent Technologies, Santa Clara, CA, USA) and an Agilent 2100 bioanalyzer, with samples having an RNA integrity number (RIN) below 7 being discarded. For sequencing, 50‐100 ng of total RNA was used to construct libraries with the VAHTS Universal V8 RNA‐seq Library Prep Kit for Illumina (NR605, Vazyme Biotech Co., Ltd., Nanjing, Jiangsu, China). PCR products were purified and size‐selected using VAHTS mRNA Capture Beads (Vazyme), and the library quality was assessed on an Agilent Bioanalyzer 2100. Libraries were then sequenced (paired‐end) on Illumina NovaSeq™ 6000 Sequencing System (Azenta, Nanjing, Jiangsu, China).

Quality control was performed using FastQC, and reads were trimmed with TrimGalore. The cleaned reads were aligned to the mm10 reference genome using STAR. Exonic reads were counted with featureCounts, and genes with zero read counts in more than one‐third of the samples were filtered out. Differential expression analysis was conducted using the DESeq2 package, with differentially expressed genes defined by BH‐corrected p‐values < 0.05. GO, KEGG and GSEA enrichment analyses were performed using ClusterProfiler.

### ATAC‐Seq Library Construction and Sequencing

As previously described,^[^ ^67^ ^]^ in a sterile PCR tube, mix 10 µL of 5× TTBL, 50 ng of DNA, 5 µL of TTE Mix V50, and ddH2O to a final volume of 50 µL. Pipette up and down 30 times to mix, then incubate at 37 °C for 45 min. Purify the fragmented DNA using the Min Elute Gel Extraction Kit (28606, Qiagen, Hilden, Germany). Enrich the purified DNA fragments by PCR for 9 cycles. Add 25 µL of mixed SPRI Beads (Beckman Coulter) to the 50 µL PCR product, mix well, and incubate at room temperature for 5 min. Centrifuge briefly and place the tube on a magnetic rack to separate the beads. Transfer the supernatant to a new sterile PCR tube. Add another 50 µL of SPRI Beads, mix well, and incubate for 5 min. After centrifuging, place the tube on a magnetic rack and remove the supernatant. Wash the beads 2‐3 times with 200 µL of 80% ethanol, incubating for 30 s each time, then gently remove the supernatant. Leave the tube open at room temperature for 5 min to dry the beads. Elute the DNA with 22 µL of sterilized ultrapure water, mix well, and incubate for 5 min. Centrifuge briefly, place the tube on a magnetic rack, and transfer 20 µL of the supernatant to a new sterile PCR tube for storage at −20 °C. Measure the library concentration with Qubit, then analyze the library on an Agilent 4200 Bioanalyzer for length and distribution.

The raw ATAC‐seq FASTQ files were first processed with FastQC to assess the quality of the reads, checking factors such as GC content, adapter contamination, and sequence quality scores. The reads were then trimmed using Trim Galore and aligned to the mm10 reference genome with Bowtie2. Subsequent processing, including binarization, sorting, deduplication, and indexing, was performed using SAMtools. Bigwig files were generated with the “bamCoverage” function of Deeptools. Peak calling was carried out using MACS2. Differential accessibility analysis was performed with DiffBind, where peaks were counted using summits = FALSE and bUseSummarizeOverlaps = TRUE. Differences were reported using method = DBA_DESEQ2, contrast = 1, and threshold = 0.05. Differential peaks were defined by FDR < 0.05 and absolute Log2FoldChange > 0.5. Genomic annotation of peaks was conducted using ChIPseeker and ClusterProfiler. Motif enrichment between the *Ogt^flox‐/Y^* and *Ogt^flox+/Y^* groups was calculated with HOMER, and transcription factor binding sites were predicted using a known motif strategy. GO/KEGG‐related genes were identified from the database, with P < 0.05 considered significant.

### Proteomics and Phosphoproteomics Sequencing

Male *Ogt^flox+/Y^* mice and their littermate controls, *Ogt^flox‐/Y^*, aged 6 to 8 weeks, were used for the study. *AAV‐TH‐Cre‐WPRE‐hGHpA* (diluted to 10^12^ IU/mL) was microinjected into the VTA. After a four‐week recovery period, brain tissue was collected under anesthesia. The VTA region (coronal sections, 2.90 mm to 3.40 mm posterior to bregma) was dissected using a stereotaxic mold and placed in a pre‐chilled 1×HBSS solution. The VTA tissue was then homogenized in lysis buffer (1% Igepal, 150 mm NaCl, 50 mm Tris‐HCl (pH 6.8), 1 mM EDTA (Sigma), 0.5 mM DTT (Thermo Fisher Scientific), Protease Inhibitors (Roche), and PhosSTOP (Roche)) using a Tissuelyzer II.^[^ ^68^ ^]^

For proteomics, the digested peptides were purified using a C18 solid‐phase extraction column to remove salts and other impurities. The purified peptides were then analyzed with a high‐resolution mass spectrometer (Orbitrap) operating in positive ion mode, where peptides were ionized by electrospray ionization (ESI) for analysis. Data‐dependent acquisition (DDA) was used to select the most intense ions for tandem mass spectrometry (MS/MS) analysis. For phosphoproteomics, phosphorylated peptides were enriched using titanium dioxide (TiO2). MS/MS analyses were performed with a high‐resolution mass spectrometer, utilizing data‐dependent acquisition (DDA).

For proteomics, MaxQuant (https://www.maxquant.org/) was used to perform peptide matching and identification based on mass spectrometry data, followed by quantification. Peptide matching was conducted by comparing the data to known protein databases such as Uniprot (https://www.uniprot.org/), allowing for the identification of proteins present in the samples. Data normalization and statistical analysis were carried out using Perseus (https://maxquant.net/perseus/) to identify differentially expressed proteins under various conditions. Functional enrichment analysis was performed using clusterProfiler.

For phosphoproteomics, data processing was also conducted with MaxQuant to identify phosphorylation sites. These sites were further validated against the PhosphoSitePlus database (https://www.phosphosite.org/). Quantitative analysis of phosphorylation levels under different conditions was performed, followed by GO and KEGG enrichment analyses to explore the biological processes and signaling pathways regulated by phosphorylation.

### Chromatin Immunoprecipitation and qPCR Analysis

The VTA tissue from *Ogt^flox+/Y^* mice and their littermate controls, *Ogt^flox‐/Y^*, was rapidly extracted, flash‐frozen, and stored at −80 °C until further processing. ChIP was performed using the Agarose ChIP Kit (Sigma–Aldrich) according to the manufacturer's instructions, with slight modifications. Briefly, the samples were minced and crosslinked with 1.5% formaldehyde at room temperature for 10 min, followed by quenching with glycine for 5 min. The samples were then homogenized and chromatin was sheared by incubating with 0.5 µl of Micrococcal Nuclease at 37 °C for 20 min, followed by sonication (100 W, 30 s on/off cycles; 10 cycles) to disrupt the nuclear membrane. For immunoprecipitation, the diluted chromatin was incubated overnight at 4 °C with 3 µl of normal IgG (Abcam, ab171870) or 3 µl of anti‐HA antibody (Sigma–Aldrich, H6908) with continuous rotation, followed by an additional 2 h incubation with 30 µl of Protein G magnetic beads. DNA was then eluted and purified from the beads and subjected to qPCR. ChIP‐qPCR results were calculated as a percentage of input DNA. All primers used for PCR amplification were listed in Table S3 (Supporting Information).

### Statistical Analysis

Data were analyzed using SPSS version 22 (IBM, Armonk, NY, USA) and MATLAB. The normality of the data was assessed with the Shapiro‐Wilk test. Between‐group comparisons were performed using a two‐tailed Student's t‐test. For comparisons across multiple groups, one‐way ANOVA, two‐way ANOVA, or two‐way repeated measures (RM) ANOVA was employed, followed by Bonferroni's post hoc test. Non‐normally distributed data were analyzed using non‐parametric tests. Statistical significance was defined as ^*^P < 0.05, ^**^P < 0.01, and ^***^P < 0.001. All data are presented as mean ± SEM. Detailed statistical information for each figure was presented in Table S2 (Supporting Information).

## Conflict of Interest

The authors declare no conflict of interest.

## Author contributions

M.S., Y.W., and H.W. contributed equally to this study. L.M. and F.W. supervised the study. M.S. contributed to the experimental design, statistical analysis, and drafting of the manuscript. M.S. and Y.W. performed the animal surgery, behavioral, and immunostaining tests. M.S. and C.Z. conducted the RNA‐seq and ATAC‐seq library construction. M.S. and W.H. performed the bioinformatic and data analysis. C.J., Q.L., Y.Z. and Y.J. provided critical consumables. L.M., F.W., and X.L. revised the manuscript.

## Supporting information



Supporting Information

Supporting Information

Supporting Information

Supporting Information

## Data Availability

The data that support the findings of this study are available from the corresponding author upon reasonable request.
