# Supplementary material for: Counterbalancing O‐GlcNAcylation and STAT3 Phosphorylation in Ventral Tegmental Area Dopaminergic Neurons Mediates Behavioral Adaptations to Acute Restraint Stress
Source: Adv Sci (Weinh). 2025 Jul 21;12(39):e02701. doi: 10.1002/advs.202502701 (PMC12533289; doi:10.1002/advs.202502701)
Supplement: Supplementary file 1 — Supporting Information [file ADVS-12-e02701-s004.pdf]

Supplementary Information

Counterbalancing O-GlcNAcylation and STAT3 Phosphorylation in Ventral Tegmental Area Dopaminergic Neurons Mediates Behavioral Adaptations to Acute Restraint Stress

Mingshuo Shao[#1,2], *Yi Wu*[#1,2], *Haiyang Wang*[#1], *Chenchun Zhang*[1], *Ying Zhu*[1], *Yan Jiang*[1], *Changyou Jiang*[1,2], *Qiumin Le*[1,2], *Xing Liu*[1,2], *Lan Ma*[1,2,] *and Feifei Wang*[*1,2]

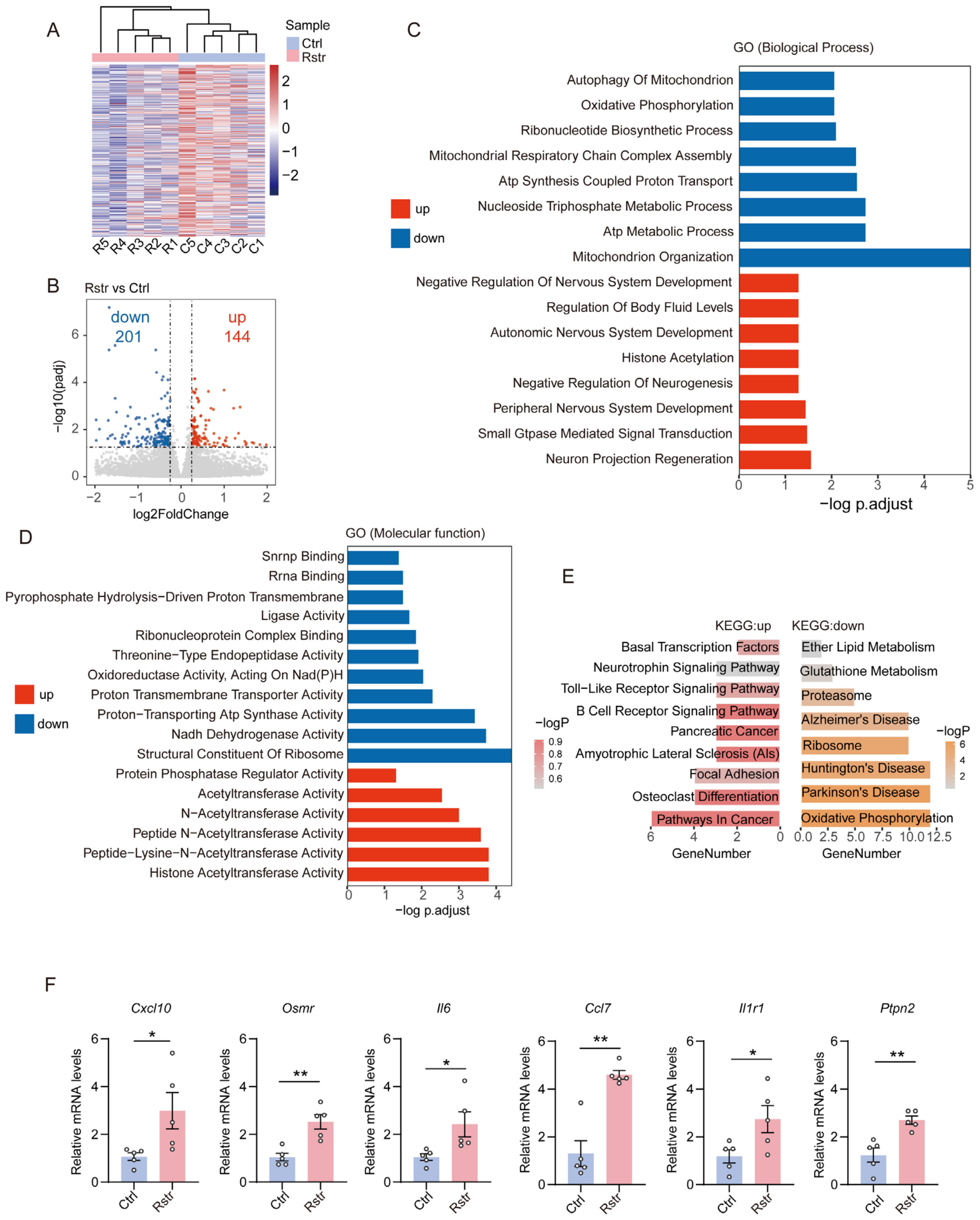

Figure S1. A comprehensive analysis of RNA-seq data from VTA DAergic neurons revealed DEGs and their associated pathways changed by acute restraint stress. A) Heatmap of differentially expressed genes in VTA DAergic neurons (DEGs: fold change > 2 and adjusted P-value < 0.05). n = 5 samples from 10 mice per group. B) Volcano plot showing DEGs in DAergic neurons in restraint and control groups. Each colored dot represents a DEG. Points with an adjusted P-value < 0.05 are highlighted.

C) GO biological process enrichment analysis of DEGs in DAergic neurons. D) GO molecular function enrichment analysis of DEGs in VTA DAergic neurons. E) KEGG pathway enrichment analysis of DEGs in VTA DAergic neurons. F) qRT-PCR analysis of the ribosome-associated mRNAs from VTA DAergic neurons. $n = 5$ Samples from 10 mice per group. Data are presented as mean ± SEM. * $p < 0.05$, ** $p < 0.01$; p-values are calculated using Mann-Whitney U test (*Ccl7*) or two-tailed unpaired Student's t-test (*Cxcl10, Osmr, Il6, Il1r1,* and *Ptpn2*).

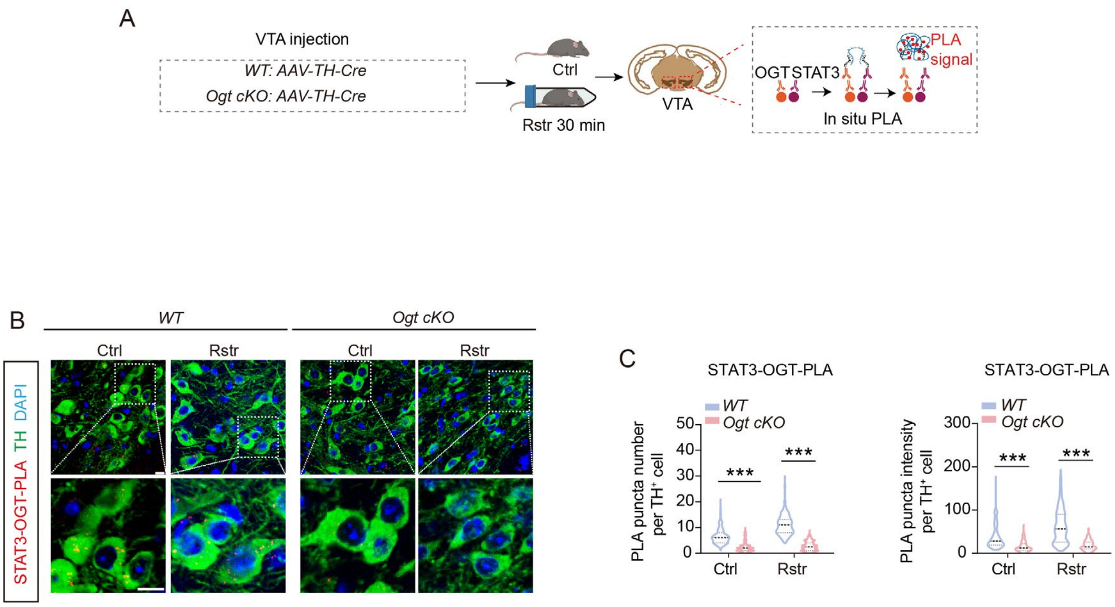

Figure S2. Knockdown of *Ogt* in VTA DAergic neurons resulted in the abolition of the PLA signal between OGT and STAT3. A–C) *AAV-TH-Cre* was injected into the VTA of *Ogt*$^{flox+/Y}$ and *Ogt*$^{flox-/Y}$ mice. Representative confocal images (A), the quantification of the number (B), and the intensity (C) of OGT-STAT3 PLA puncta per TH^{+} cell in the VTA DAergic neurons from *Ogt*$^{flox+/Y}$ and *Ogt*$^{flox-/Y}$ groups. n = 240 cells from 6 mice per group. Green: TH; Red: PLA puncta; Blue: DAPI. Scale bar: 20 µm. Data are presented as mean ± SEM. ***p < 0.001; p-values are calculated using Scheirer-Ray-Hare test with *Bonferroni post hoc* test (C).

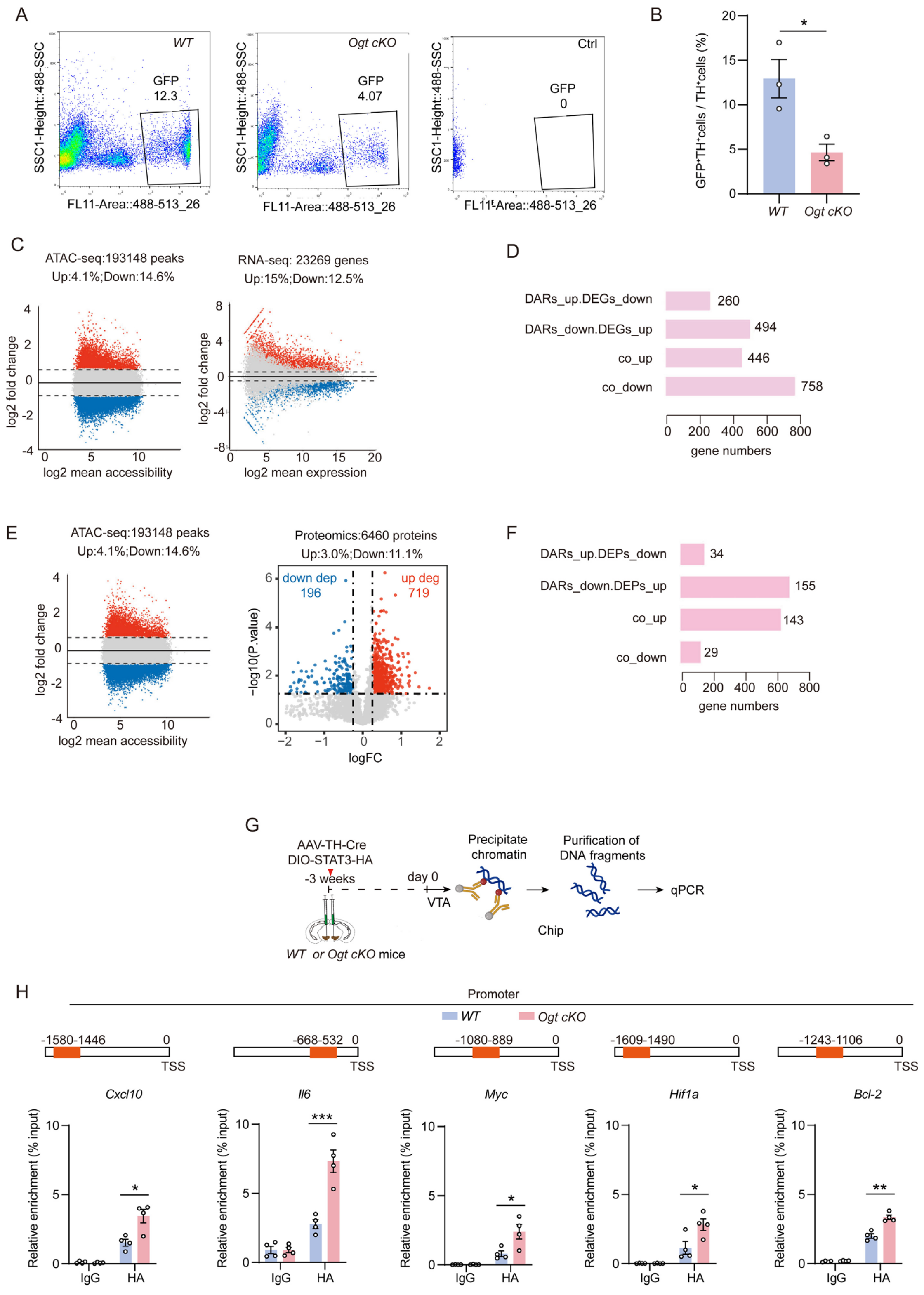

Figure S3. ATAC-seq, RNA-seq, and proteomics analysis to investigate the effect of *Ogt* knockdown in VTA DAergic neurons. A–B) Representative flow cytometry sorting images (A) and quantitative analysis (B) of GFP^{+} neuron numbers in the VTA from *Ogt*$^{flox+/Y}$ and *Ogt*$^{flox-/Y}$mice. n = 3 samples from 9 mice per group. GFP^{+}

populations are highlighted with black boxes. C) MA plots showing DARs and DEGs in DAergic neurons after *Ogt* knockdown. Each dot represents a peak region or gene, with colored dots indicating DARs or DEGs. D) Genes overlapping with DARs and DEGs were categorized into four groups based on expression changes: DARs_up & DEGs_down, DARs_down & DEGs_up, co_up, and co_down. E) MA and volcano plots illustrating DARs and DEPs in DAergic neurons following *Ogt* knockdown. Each dot represents a peak region or protein, with colored dots indicating DARs or DEPs F) Genes overlapping with DARs and DEPs were categorized into four groups: DARs_up & DEPs_down, DARs_down & DEPs_up, co_up, and co_down. G) Schematic diagram illustrating the STAT3-specific ChIP assay performed on VTA tissue. *AAV-TH-Cre* and Cre-dependent STAT3-HA were injected into the VTA of $Ogt^{flox+/Y}$ and $Ogt^{flox-/Y}$ mice. H) qRT-PCR analysis of the enrichment of STAT3 at genomic regions upstream of the TSS of *Cxcl10, Il6, Myc, Hif1a, and Bcl-2*, as determined by HA-specific ChIP. IgG was used as a negative control. The enrichment was normalized to the input. $n = 4$ samples from 12 mice per group. Data are presented as mean ± SEM. * $p < 0.05$, ** $p < 0.01$, *** $p < 0.001$; *p*-values are calculated using Two-tailed unpaired Student's t-test (B) or Two-way RM ANOVA with *Bonferroni post hoc* test (H).

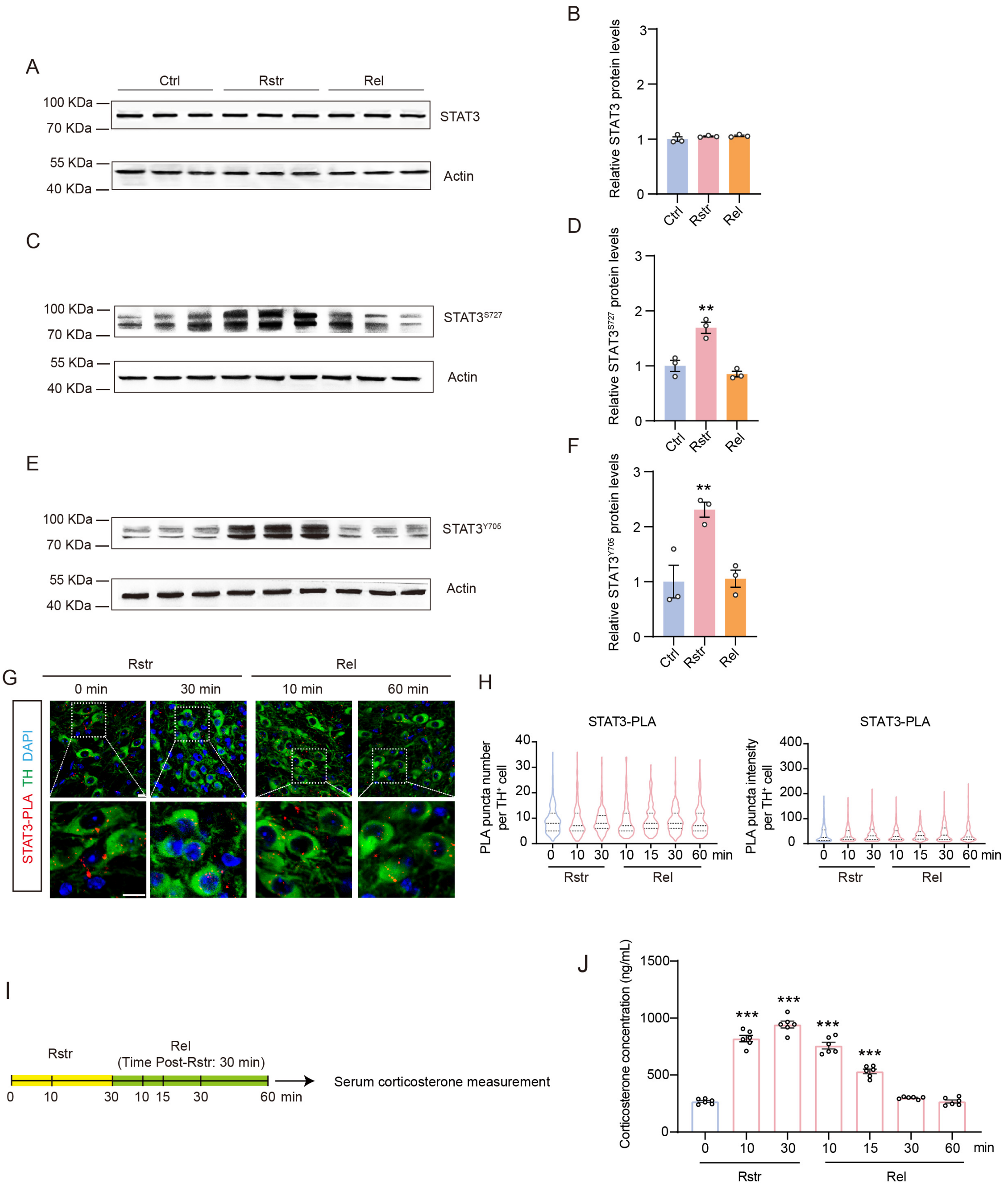

Figure S4. Acute restraint stress increased STAT3 phosphorylation in the VTA and elevated serum corticosterone levels. A–B) Representative immunoblots (A) and quantification of the STAT3 protein level (normalized to actin) (B). C–D) Representative immunoblots (C) and quantification of the phosphorylated STAT3^{S727} protein level (normalized to actin) (D). E–F) Representative immunoblots (E) and quantification of the phosphorylated STAT3^{Y705} protein level (normalized to actin) (F) are shown. n = 3 samples from 6 mice per group. G–H) Representative confocal images (G) and quantitative analysis of the mean number and intensity (H) of PLA puncta per

TH^+ cell. Green: TH; Red: PLA puncta of STAT3; Blue: DAPI. Scale bar: 20 μm. n = 240 cells from 6 mice per group. I–J) Timeline schematic of serum corticosterone measurement after acute restraint stress (10- or 30-min restraint, with sampling at 10-, 15-, 30-, and 60-min post-release) (I), and quantification of serum corticosterone levels at the indicated time points (J). n =6 mice per group. Data are presented as mean ± SEM.** $p < 0.01$, *** $p < 0.001$; p-values are calculated using One-way ANOVA (B, D, J), Welch's ANOVA (F) with *Bonferroni post hoc* test. Kruskal–Wallis H test with *Bonferroni post hoc* test (H).

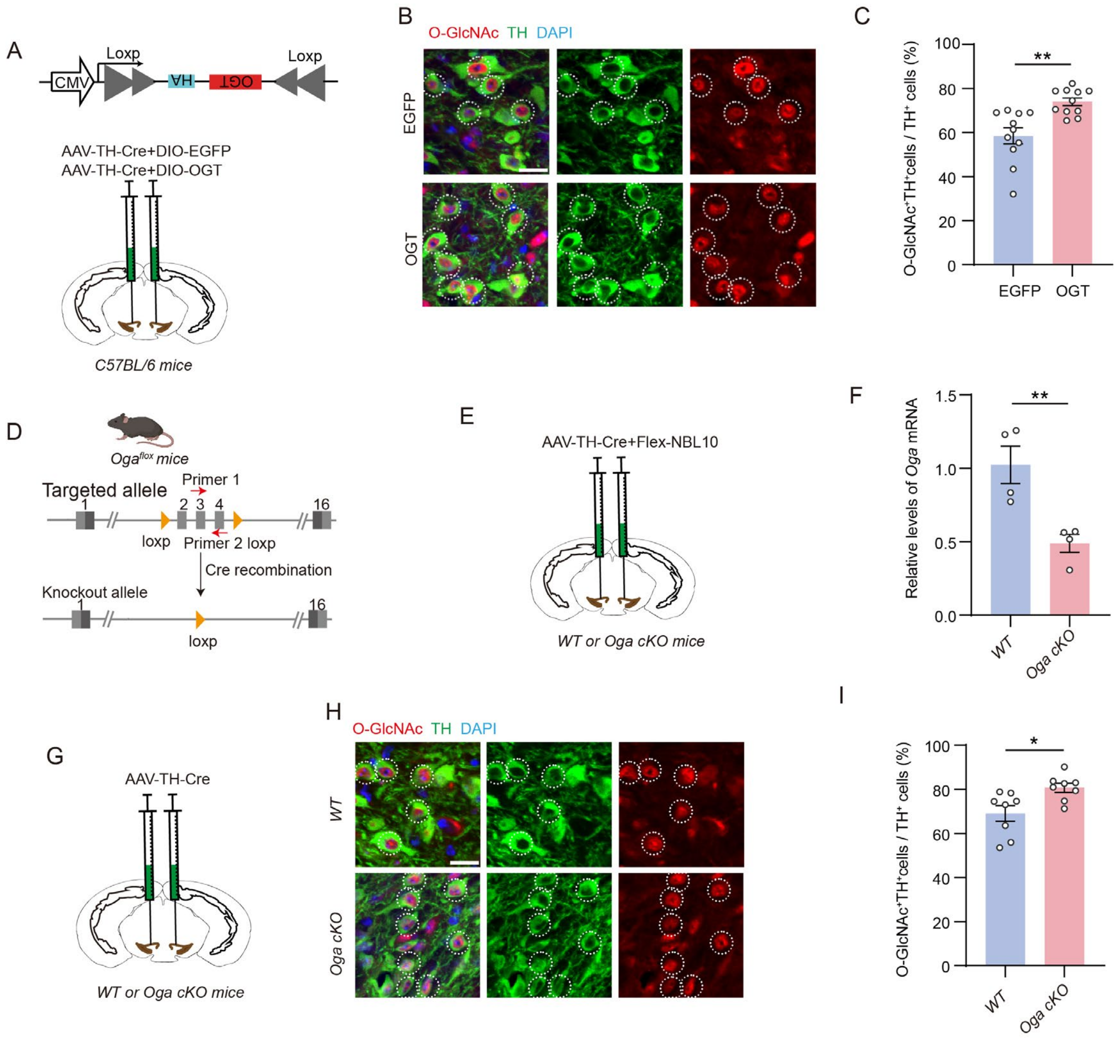

Figure S5. AAV constructs encoding Cre-dependent OGT and the generation of *Oga cKO* mice. A) Schematic diagram illustrating AAV encoding Cre-dependent OGT construct with an HA tag and the viral injection strategy. B–C) Representative confocal images (B) and quantification (C) of the proportion of O-GlcNAc^{+} cells in VTA TH^{+} neurons. n = 11 mice per group. Green: TH; Red: O-GlcNAc; Blue: DAPI. Scale bar: 20 μm. D) Diagram of the targeting strategy for generating *Oga* cKO mice. Red arrows indicate the primers used to identify the floxed sites in *Oga* cKO mice, located between exons 3 and 4. E) *AAV-TH-Cre* and *AAV-Flex-NBL10* were injected into the VTA of *Oga*$^{flox+/+}$ and *Oga*$^{flox-/-}$mice. F) qRT-PCR analysis of *Oga* mRNA in the ribosome-associated mRNAs of VTA TH^{+} cells from the *Oga*$^{flox+/+}$ and *Oga*$^{flox-/-}$ groups. n = 4 samples from 8 mice per group. G) *AAV-TH-Cre* was injected into the VTA of *Oga*$^{flox+/+}$ and *Oga*$^{flox-/-}$mice. H–I) Representative confocal images (H) and quantification (I) of

the proportion of O-GlcNAc$^+$ cells in TH$^+$ neurons in *Oga*$^{flox+/+}$ and *Oga*$^{flox-/-}$mice. n = 8 mice per group. Green: TH; Red: O-GlcNAc; Blue: DAPI. Scale bar: 20 µm. Data are presented as mean ± SEM. * $p < 0.05$, ** $p < 0.01$; p-values are calculated using Two-tailed unpaired Student's t-test (C, I). Welch's t-test (F).

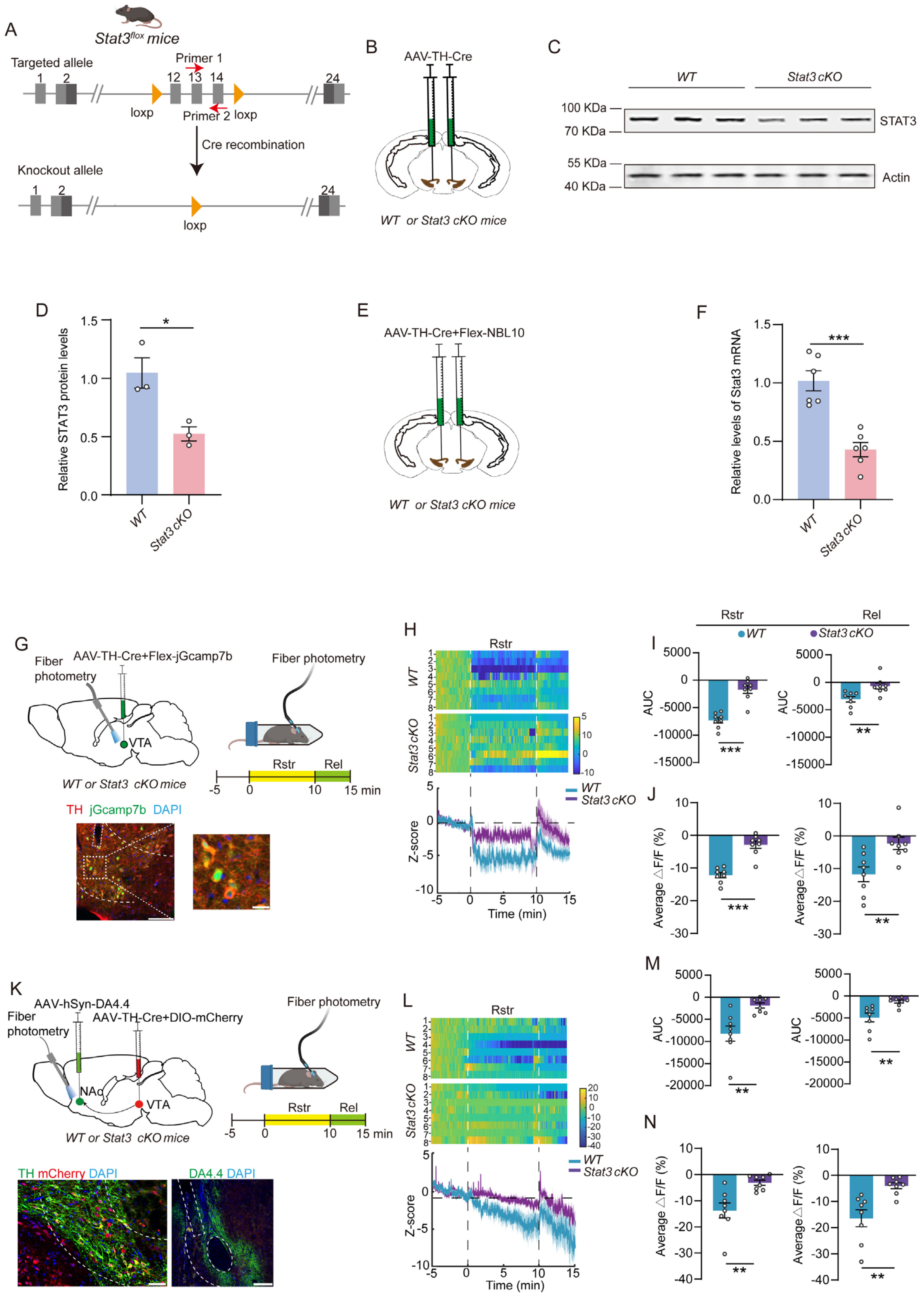

Figure S6. The suppression of neuronal activity induced by acute restraint stress was abolished by the *Stat3* knockdown in VTA DAergic neurons.

A) Diagram illustrating the targeting strategy for generating *Stat3 cKO* mice. Red arrows indicate primers used to identify the floxed sites located between exons 13 and 14. B–D) Validation of *Stat3* knockdown effects by Western blot. (B) *AAV-TH-Cre* was injected into the VTA of *Stat3 cKO* mice to knock down *Stat3* in the VTA TH^{+} cells. (C–D) Representative immunoblots (C) and quantification of STAT3 protein levels (normalized to actin) (D). $n = 3$ samples from 6 mice per group. E–F) Validation of *Stat3* knockdown effects by qRT-PCR. (E) *AAV-TH-Cre* and *AAV-Flex-NBL10* were injected into the VTA of $Stat3^{flox+/+}$ and $Stat3^{flox-/-}$mice. (F) qRT-PCR analysis of *Stat3* mRNA in the ribosome-associated mRNAs of TH^{+} cells. $n = 6$ samples from 12 mice per group. G–J) Calcium signal recording of VTA DAergic neurons during acute restraint stress. (G) Schematic diagram depicting viral injection and fiber photometry recording. Representative images show jGCaMP7b expression and fiber placement in the VTA. Green: jGCaMP7b; Red: TH; Blue: DAPI. Scale bar: left, 100 μm; right, 20 μm. Dashed white lines outline the fiber optic tract, and the dashed box indicates enlarged areas. (H–J) Heat maps (top) and Z-score normalized average calcium signals (bottom) for restraint and release conditions. $n = 8$ mice per group. Shadows represent SEM. Quantification of area under the curve (AUC) (I) and Average peak calcium signal responses (J) aligned to the onset of restraint. K–N) DA signal recording in the NAc using the DA4.4 sensor during acute restraint stress. (K) Schematic diagram illustrating viral injection and fiber photometry recording. Representative images show mCherry expression in the VTA and DA4.4 expression in the NAc. Green: TH/DA4.4; Red: mCherry; Blue: DAPI. Scale bar: left, 50 μm; right, 100 μm. Dashed white lines outline the brain region and fiber optic tract. (L–N) Heat maps (top) and Z-score normalized average peak of DA4.4 signals (bottom) for restraint and release conditions. $n = 8$ mice per group. Shadows represent SEM. Quantification of AUC (M) and Average peak of DA4.4 responses (N) aligned to the onset of restraint and release. Data are presented as mean ± SEM. * $p < 0.05$, ** $p < 0.01$, *** $p < 0.001$; p-values are calculated using Two-tailed unpaired Student's t-test (D, F, I, J, M, N) or Mann-Whitney U test (N).

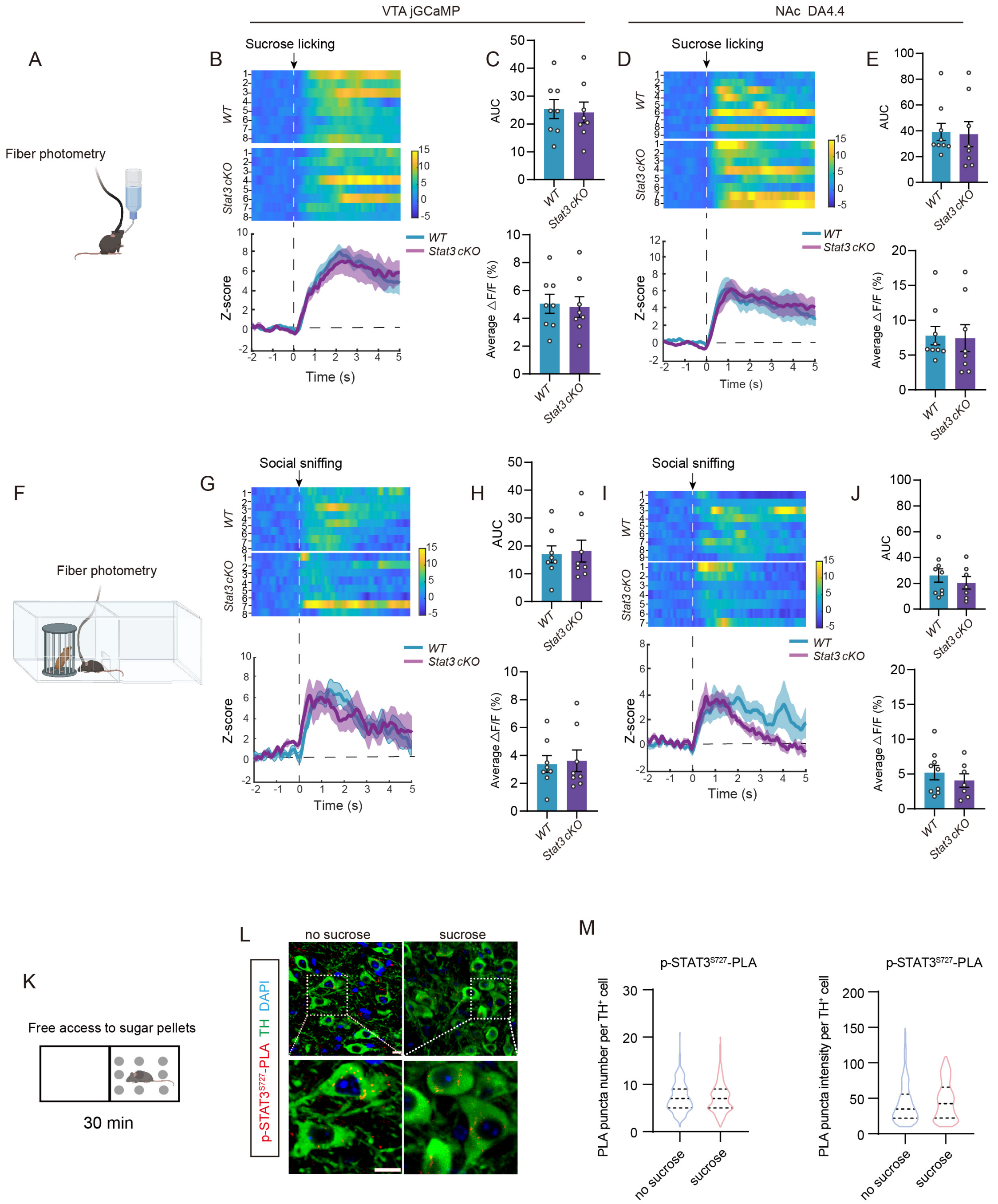

Figure S7. Neuronal activity or DA release in response to natural rewards was not affected by *Stat3* knockdown in VTA DAergic neurons. A) Schematic diagram illustrating fiber photometry recording of calcium signal in the VTA and DA release in the NAc during the sucrose water licking in freely moving mice. B, D) Heat maps (top) and average calcium (B) and dopamine sensor (D) signal responses (bottom) aligned to the onset of sucrose licking. The average response for the first five licks was calculated. Shadows represent SEM. C, E) Quantification of the area under the curve (AUC, left)

and Z-score normalized peak calcium signal (right) of jGCaMP (C) and DA4.4 (E) signals aligned to the onset of sucrose licking. For jGCaMP: n = 8 mice per group; For DA4.4: n = 8 mice in $Stat3^{flox+/+}$ group; n = 9 mice in $Stat3^{flox-/-}$ group. F) Schematic diagram illustrating fiber photometry recording of calcium signal in VTA and DA release in the NAc during the sniffing in freely moving mice. G, I) Heat maps (top) and average calcium (G) and DA sensor (I) signal responses (bottom) aligned to the onset of social sniffing. The average response for the first five sniffing events was calculated. Shadows represent SEM. H, J) Quantification of AUC (left) and Z-score normalized peak calcium signal (right) of jGCaMP (H) and DA4.4 (J) signals aligned to the onset of social sniffing. n = 7 mice in $Stat3^{flox+/+}$ group; n = 9 mice in $Stat3^{flox-/-}$ group. K) Schematic diagram illustrating mice consuming sucrose pellets freely for 30 minutes. L–M) Representative confocal images (L) and quantitative analysis of number (M) and intensity (M) of p-STAT3^{S727} in TH^{+} cells. Green: TH; Red: PLA puncta; Blue: DAPI. Scale bar: 20 µm. n = 240 cells from 6 mice per group. Data are presented as mean ± SEM. p-values are calculated using Mann-Whitney U test (E, H, M) or two-tailed unpaired Student's t-test (C, E, J).

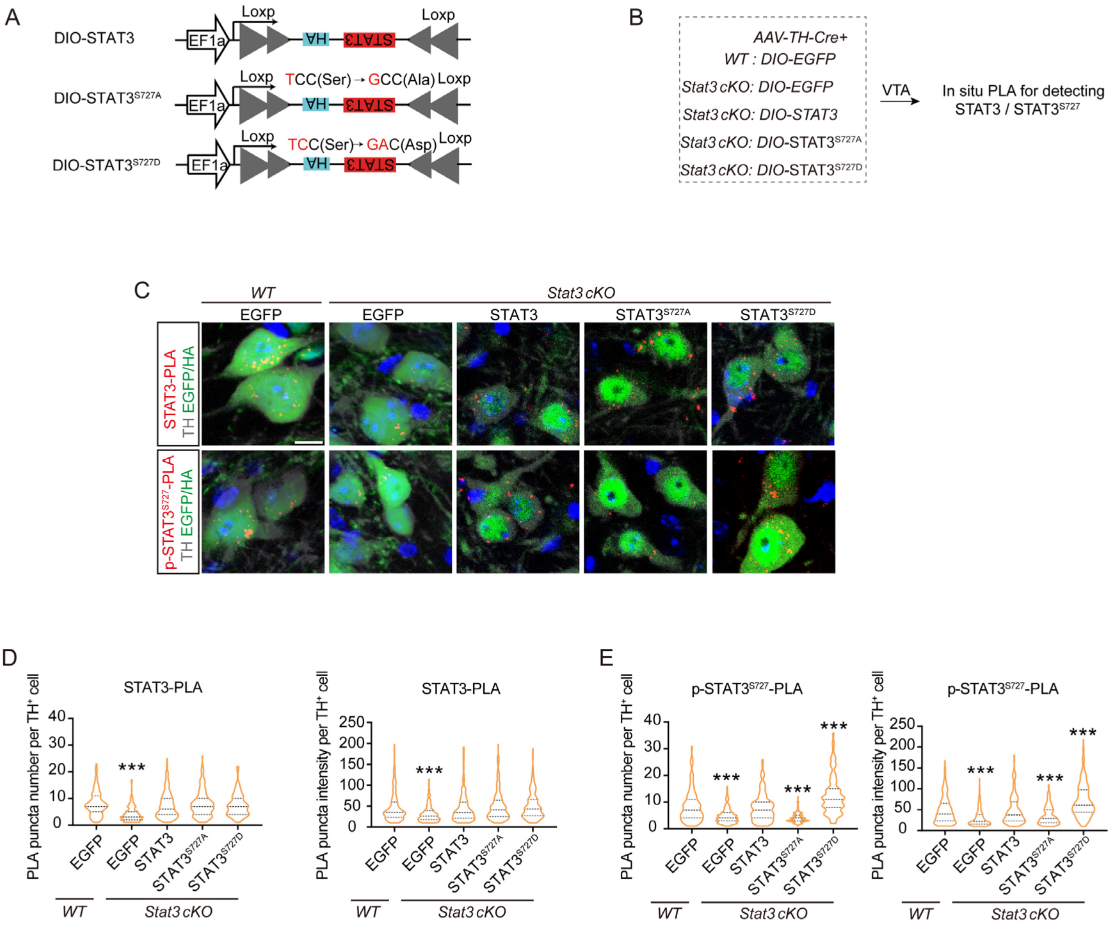

Figure S8. The validation of Cre-dependent AAV constructions encoding Stat3^{S727A} and Stat3^{S727D} mutants. A) Schematic diagram illustrating AAV constructs encoding Cre-dependent *Stat3*, *Stat3^{S727A}*, and *Stat3^{S727D}* mutants. B) The virus injection strategy. *AAV-TH-Cre* and Cre-dependent EGFP or Stat3 constructs were injected into the VTA of *Stat3$^{flox+/+}$* mice or their WT littermates. P-STAT3^{Ser727} and total STAT3 in VTA DAergic neurons were detected by PLA assay. C–E) Representative confocal images (C) and quantitative analysis of mean number (D) and mean intensity (E) of PLA puncta in TH^{+} cells. Grey: TH; Green: EGFP/HA; Red: PLA puncta of STAT3 or p-STAT3^{Ser727}; Blue: DAPI. Scale bar: 20 μm. n = 200-240 cells from 5-6 mice per group. Data are presented as mean ± SEM. *** $p < 0.001$; p-values are calculated using Kruskal–Wallis H test with *Bonferroni post hoc* test (D, E).

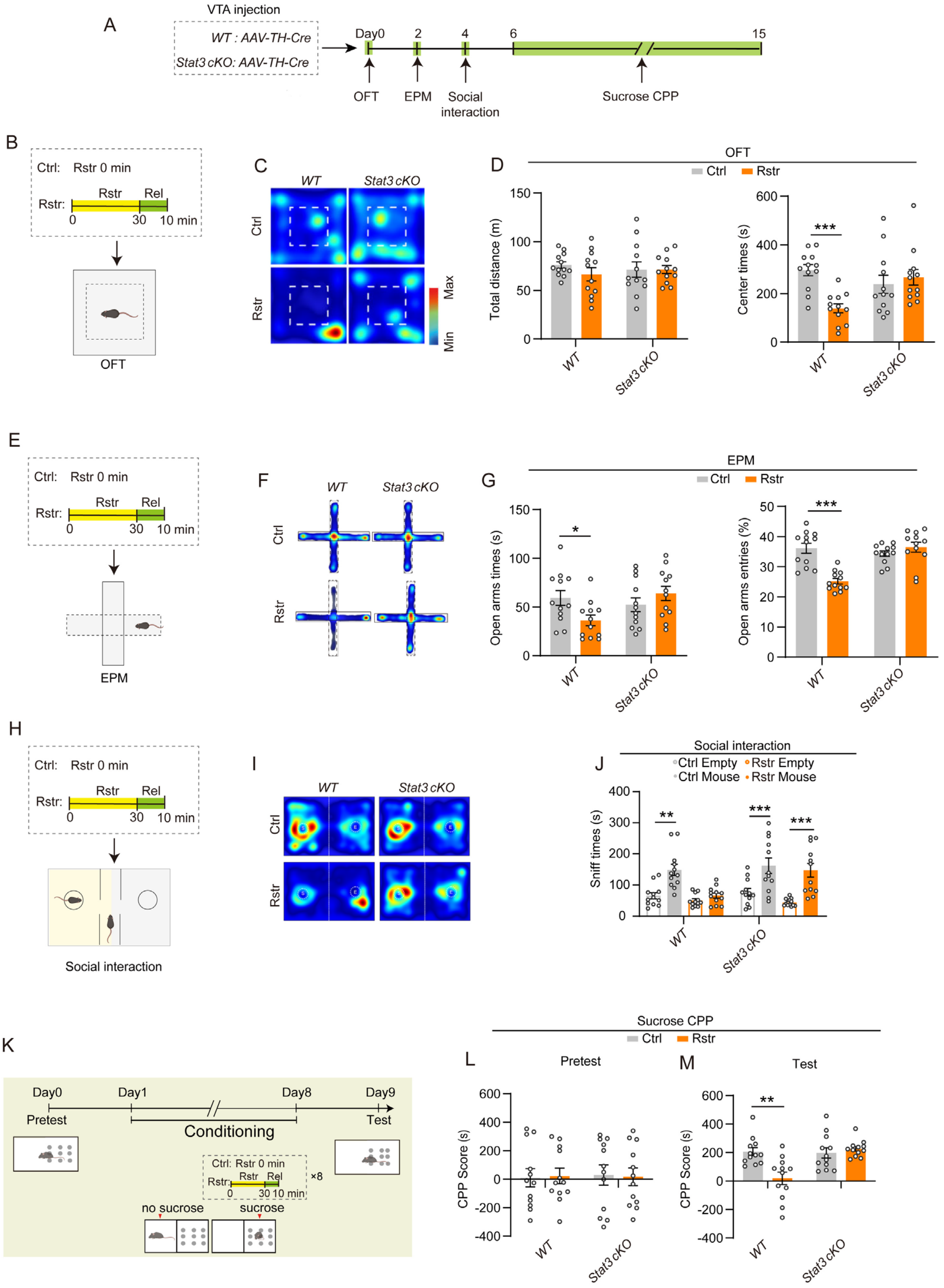

Figure S9. Knockdown of *Stat3* in VTA DAergic neurons abolished the suppression of reward sensitivity and anxiety induced by acute restraint stress. A) Schematic diagram of virus injection and the process of behavioral testing. *AAV-TH-Cre* was injected into the VTA of *Stat3 cKO* mice and their WT littermates. B–D) OFT.

Experimental diagram of OFT (B), representative heatmaps of behavioral trajectory (C) and quantitative analysis of total distance traveled and time spent in the center area (D) in mice in restraint and control groups. E–G) EPM. Experimental diagram of EPM (E), representative heatmaps of behavioral trajectory (F) and quantitative analysis of time spent and entries into open arms (G) in mice in restraint and control groups. H–J) Social interaction tests. Experimental diagram (H), representative heatmaps of behavioral trajectory (I) and quantitative analysis of the time exploring a cage containing a stranger or an empty cage (J) in restraint and control groups. K–M) Sucrose CPP. Experimental diagram (K), CPP scores during the pretest (L) and test (M) sessions. Mice underwent 30 minutes of acute restraint 10 minutes before each sucrose conditioning session. n = 12 mice per group. Data are presented as mean ± SEM. * $p < 0.05$, ** $p < 0.01$, *** $p < 0.001$; p-values are calculated using Two-way ANOVA with *Bonferroni post hoc* test (D, G, J, L, M) or Scheirer-Ray-Hare Test with *Bonferroni post hoc* test (D).

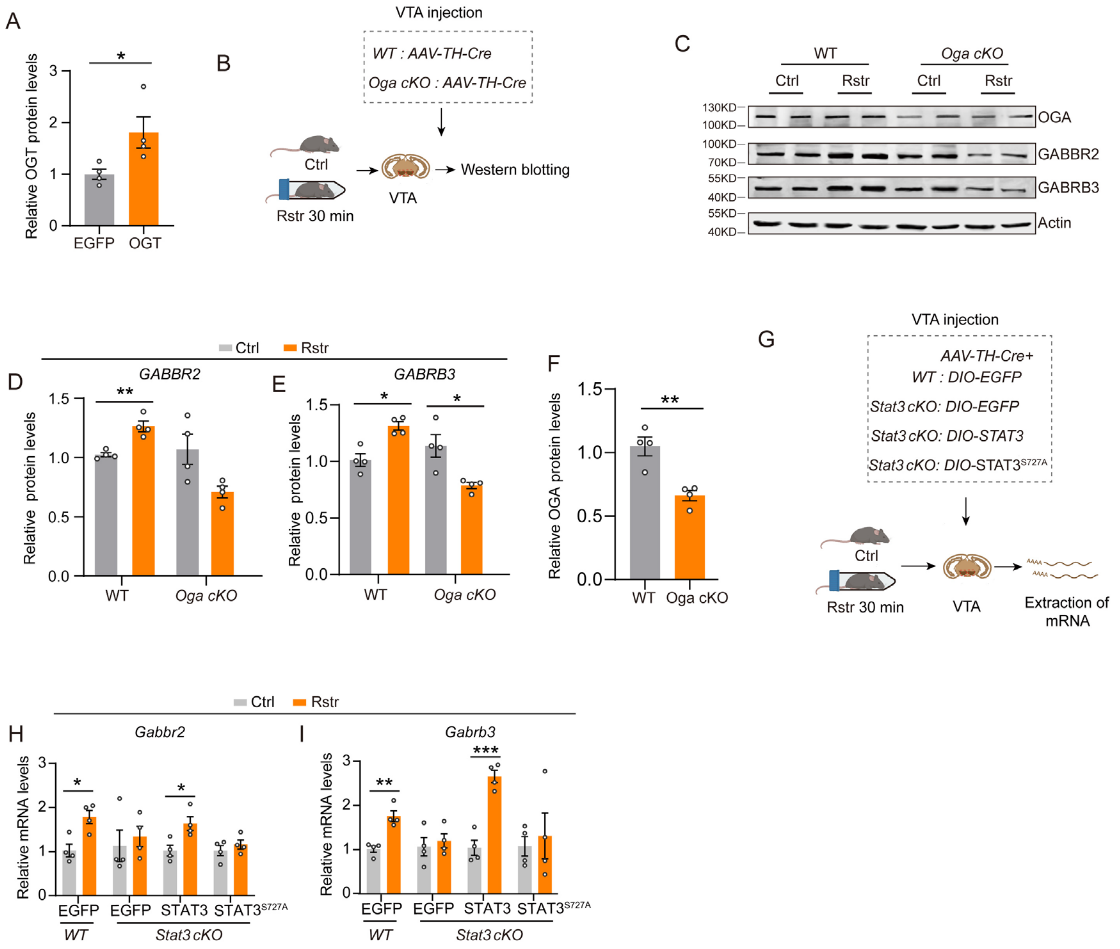

Figure S10. GABA receptor upregulation in VTA DAergic neurons after acute restraint stress is associated with O-GlcNAcylation and STAT3^{Ser727} phosphorylation. A) Quantification of OGT protein levels in VTA DAergic neurons of WT mice overexpressing EGFP or OGT.*n* = 4 samples from 8 mice per group. B) Schematic diagram of the western blot experiment. AAV-TH-Cre was injected into the VTA of *Oga*$^{flox-/-}$ or *Oga*$^{flox+/+}$ mice. VTA tissues were collected 30 minutes after acute restraint stress. C–E) Representative immunoblots (C) and quantification of GABBR2 (D) and GABRB3 (E) protein levels in the VTA, normalized to actin. *n* = 4 samples from 8 mice per group. F) Quantification of OGA protein levels in VTA DAergic neurons of *Oga*$^{flox-/-}$ and *Oga*$^{flox+/+}$ mice. G) Schematic diagram of detection of *Gabbr2* and *Gabrb3* transcripts in the VTA DAergic neurons by qRT-PCR. *AAV-TH-Cre, Flex-NBL10,* and Cre-dependent constructs encoding EGFP, STAT3, or STAT3^{Ser727A} were injected into the VTA of *Stat3*$^{flox+/+}$ or *Stat3*$^{flox-/-}$ mice. H-I) qRT-PCR analysis of *Gabbr2* and *Gabbr3* transcripts in VTA DAergic neurons from mice with or without acute

restraint stress. $n = 4$ samples from 8 mice per group. Data are presented as mean ± SEM. * $p < 0.05$, ** $p < 0.01$, *** $p < 0.001$; p-values are calculated using Mann-Whitney U test (H), two-tailed unpaired Student's t-test (A, F, H, I), Welch's ANOVA (D) or Two-way ANOVA with *Bonferroni post hoc* test (E).
